# Cardiac and Neurological Complications Post COVID-19 Vaccination: A Systematic Review of Case Reports and Case Series

**DOI:** 10.3390/vaccines12060575

**Published:** 2024-05-24

**Authors:** Kai Wei Lee, Sook Fan Yap, Syafinaz Amin-Nordin, Yun Fong Ngeow

**Affiliations:** 1 Department of Medical Microbiology, Faculty of Medicine and Health Sciences, Universiti Putra Malaysia, Serdang 43400, Selangor, Malaysia; lee_kai_wei@yahoo.com (K.W.L.); syafinaz@upm.edu.my (S.A.-N.); 2 Department of Pre-Clinical Sciences, M. Kandiah Faculty of Medicine and Health Sciences, Universiti Tunku Abdul Rahman, Kajang 43200, Selangor, Malaysia; ngeowyf@utar.edu.my; 3 Dr. Wu Lien-Teh Centre of Research in Communicable Diseases, Universiti Tunku Abdul Rahman, Kajang 43200, Selangor, Malaysia

**Keywords:** vaccines, complications, cardiac, neurological, public health, sequelae

## Abstract

Following mass vaccinations for the control of the COVID-19 epidemic, a spectrum of cardiac and neurological disorders was reported among vaccinated individuals. This study examined the range of complications documented and factors related to their occurrence. Three electronic databases were searched for case reports and case series with descriptions of cardiac and/or neurological complications in COVID-19 vaccine recipients. A total of 698 vaccinees were included in this review, of which 259 (37.1%) had cardiac and 439 (62.9%) had neurological complications. Inflammatory conditions were the commonest among the cardiac complications; while polyneuropathy, demyelinating diseases and cerebrovascular disorders were the more common neurological complications. The mean age of those with cardiac complications (33.8 years) was much younger than those with neurological complications (49.7 years). There was no notable difference in the gender distribution between these two groups of vaccine recipients. mRNA vaccines (all brands) were associated with almost 90.0% of the cardiac complications, whereas viral vector vaccines were associated with slightly over half (52.6%) of the neurological complications. With regard to the dose, cardiac complications were more common after the second (69.1%), whereas neurological complications were more common after the first dose (63.6%). The majority of the cases had an uncomplicated clinical course. Nevertheless, 5.9% of cases with neurological complications and 2.5% of those with cardiac complications were fatal, underscoring the significance of the consistent surveillance and vigilant monitoring of vaccinated individuals to mitigate these occurrences.

## 1. Introduction

The COVID-19 pandemic caused catastrophic medical and public health problems worldwide. Urgent control measures were needed to stem its onslaught, the most effective of which was mass vaccination to prevent infection with the SARS-CoV2 virus. As a result, many vaccines of various designs were developed, and a universal mass vaccination program began in late 2021.

The application of mass vaccination includes the description and reporting of adverse side effects or complications associated with the use of the vaccines. As publicity on vaccine-related complications can adversely affect vaccine uptake, it is essential that information on these complications be scientifically validated.

Early clinical data from various trials indicated that approved COVID-19 vaccines were safe for healthy adults. Nevertheless, complications following the administration of approved vaccines have been reported worldwide [1]. These complications affected different organ systems, two of the most widely recognized being the cardiovascular and nervous systems. The most common cardiac complication reported was myocarditis [2], while the neurological complications most often reported included Guillain–Barré syndrome (GBS) [3], transverse myelitis [4] and Bell palsy [5].

This systematic review was undertaken to compile the spectrum of cardiac and neurological complications reported in vaccine recipients and to study their association with demographic, health and vaccination-related factors.

## 2. Materials and Methods

### 2.1. Protocol Registration

This review was conducted according to the Preferred Reporting Items for Systematic Reviews and Meta-Analyses (PRISMA) (Supplementary S1) [6]. It has been registered with PROSPERO (Registration number: CRD42022310861).

### 2.2. Literature Search

The search for articles began on 14 February 2022, using the following bibliographic databases: PubMed, Medline and Scopus. The search terms used were (COVID-19 vaccine) AND (Safety OR adverse event OR adverse reaction OR side-effect OR complication OR manifestation OR impairment OR disorder OR disease) AND (Nervous system OR neurological) OR (Cardiovascular OR heart) NOT (review OR systematic review OR meta-analysis). No language or geographical area restriction was imposed on the eligibility for inclusion; however, articles had to be published in the years 2021 or 2022. The findings from each search are tabulated in Supplementary S2. Reverse and forward citation tracking was performed to seek any relevant papers that were missed during the initial search. The last search was performed on 25 June 2022. Papers published beyond this date were not considered for inclusion.

### 2.3. Data Handling

All relevant articles identified through the databases were imported into the Endnote Programme, version X5. Duplications were removed, articles were screened by their title, and abstracts and papers were subjected to full-text assessment for inclusion. These steps were conducted independently by KWL and SFY. Discrepancies between the two investigators were resolved through discussion with the third and fourth investigators (SAN and YFN) to reach a final consensus.

### 2.4. Selection Criteria

The types of studies eligible for inclusion were case reports and case series. Only studies with confirmed diagnoses of cardiac and/or neurological complications post COVID-19 vaccination were selected. Cases with a history of COVID-19 were excluded as the infection is widely known to cause cardiac and neurological complications. Cases with unconfirmed or uncertain diagnoses were also excluded.

### 2.5. PICO

The participants were those who received at least one dose of the COVID-19 vaccine and had no history of COVID-19. Exposure referred to COVID-19 vaccine recipients who presented with cardiac or/and neurological complications post vaccination. There was no comparator in the current review. The main outcome of this review was a summary of the spectrum of cardiac and neurological complications post COVID-19 vaccination.

### 2.6. Data Extraction

The relevant information summarized in the Results Section included the basic characteristics of the studies (i.e., author names, year of publication, country of study), patient demographic characteristics (age and sex) and their vital signs (blood pressure, pulse rate, respiration rate, oxygen saturation at ambient temperature) at hospital admission. The following supplemental information was included and reviewed for the evaluation of their diagnoses: (i) medical history and comorbidities, (ii) clinical presentation, (iii) results of clinical examination and laboratory investigations (in vitro and in vivo) and (v) treatment given. Also examined were the in-hospital clinical outcomes and, where available, patient condition at follow-up. The other information extracted included vaccination profiles (vaccine types and the number of doses received) and the timing of the vaccination. Detailed descriptions of the included studies are summarized in Supplementary S3 for cardiac complications and Supplementary S4 for neurological complications.

### 2.7. Data Analyses

Statistical analyses were performed using IBM SPSS Statistics version 22. The data were presented as frequencies and percentages for categorical variables and as means ± standard deviations for continuous variables. No statistical significance tests were conducted, as the analyses were primarily descriptive in nature.

## 3. Results

### 3.1. Search Results

**Figure 1:**
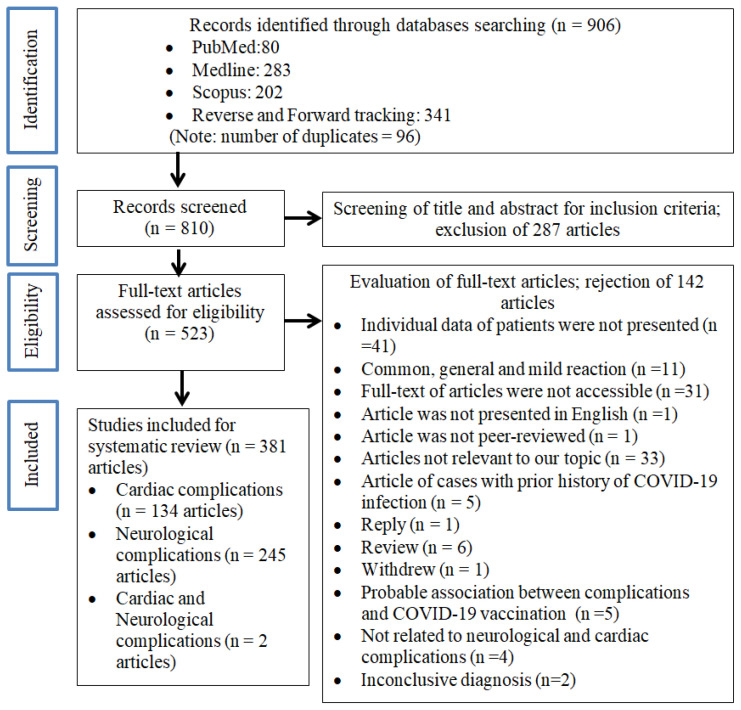
PRISMA flow diagram of the literature screening process.

Our initial search of the databases identified 906 articles (Figure 1); after removing duplicates (n = 96), 810 articles were retrieved for further assessment. After screening the titles and abstracts for suitability, 523 articles were selected for full-text assessment. Following a more detailed evaluation, 381 articles were included for systematic review. Of these, 134 articles (259 cases) reported cardiovascular complications [2,7,8,9,10,11,12,13,14,15,16,17,18,19,20,21,22,23,24,25,26,27,28,29,30,31,32,33,34,35,36,37,38,39,40,41,42,43,44,45,46,47,48,49,50,51,52,53,54,55,56,57,58,59,60,61,62,63,64,65,66,67,68,69,70,71,72,73,74,75,76,77,78,79,80,81,82,83,84,85,86,87,88,89,90,91,92,93,94,95,96,97,98,99,100,101,102,103,104,105,106,107,108,109,110,111,112,113,114,115,116,117,118,119,120,121,122,123,124,125,126,127,128,129,130,131,132,133,134,135,136,137,138,139], 245 articles (439 cases) reported neurological complications [3,140,141,142,143,144,145,146,147,148,149,150,151,152,153,154,155,156,157,158,159,160,161,162,163,164,165,166,167,168,169,170,171,172,173,174,175,176,177,178,179,180,181,182,183,184,185,186,187,188,189,190,191,192,193,194,195,196,197,198,199,200,201,202,203,204,205,206,207,208,209,210,211,212,213,214,215,216,217,218,219,220,221,222,223,224,225,226,227,228,229,230,231,232,233,234,235,236,237,238,239,240,241,242,243,244,245,246,247,248,249,250,251,252,253,254,255,256,257,258,259,260,261,262,263,264,265,266,267,268,269,270,271,272,273,274,275,276,277,278,279,280,281,282,283,284,285,286,287,288,289,290,291,292,293,294,295,296,297,298,299,300,301,302,303,304,305,306,307,308,309,310,311,312,313,314,315,316,317,318,319,320,321,322,323,324,325,326,327,328,329,330,331,332,333,334,335,336,337,338,339,340,341,342,343,344,345,346,347,348,349,350,351,352,353,354,355,356,357,358,359,360,361,362,363,364,365,366,367,368,369,370,371,372,373,374,375,376,377,378,379,380,381,382,383], and 2 articles described concurrent cardiac and neurological complications [384,385].

### 3.2. Geographical Distribution of Included Studies

The 134 articles related to cardiac complications were mostly from the USA (n = 40) and Japan (n = 20). Smaller numbers (1–10 each) were from 33 other countries in Europe, Africa, Asia, Australasia and S. America (Table 1).

The 245 reports on neurological complications were also mostly from the USA (n = 43), with 10–27 articles each from India, Italy, Japan, the UK, Iran and South Korea, and 1–9 each from another 37 countries in Europe, Africa, Asia, Australasia and S. America (Table 2).

There were two case reports from Bangladesh describing concurrent cardiac and neurological complications.

### 3.3. Characteristics of Patients

The 259 patients (139 male, 120 female; Table 1) with cardiovascular complications were aged between 12 and 96 years old. They were mostly from S. America (41.3%), Asia (38.6%) and Europe (18.9%), with just a small number from Australasia (0.8%) and Africa (0.4%). About a third (80; 30.9%) had pre-existing morbidities or a history of health problems that may or may not be risk factors for cardiac complications. The majority (233; 90%) received mRNA vaccines, 22 (8.5%) received viral vector vaccines, and the remaining 4 (1.5%) received inactivated vaccines.

The 439 patients (230 females, 200 males, 9 unknown gender; Table 2) with neurological complications were aged from 15 to 90 years old. They were from Asia (44.4%), Europe (29.6%), Latin Americas (22.6%), Australasia (3%) and Africa (0.5%). Less than half (40.5%) had pre-existing morbidities or a history of health problems that may or may not be risk factors for neurological complications. Slightly more than half (53.6%) received viral vector vaccines, 42.1% received mRNA vaccines, and a small number (4.4%) received inactivated vaccines.

Specific details on the vaccine doses and types related to complications are summarised in Supplementary S3 and S4.

### 3.4. Complications and Outcomes Post COVID-19 Vaccination

Most (86.6%) cases with cardiac complications had an uncomplicated clinical course with a fatality rate of 2.4% (Table 1). The most frequent diagnosis was inflammation of the heart (n = 221, 85.3%). Others were acute myocardial infarction including ST-elevated myocardial infarction (STEMI) and non-STEMI (n = 15, 5.8%), cardiomyopathy (n = 11, 4.2%), myocardial injury, nature not specified (n = 9, 3.5%) and cardiac arrhythmias (n = 3, 1.2%). Among those with inflammation of the heart, most had myocarditis (n = 152), followed by myopericarditis (n = 38), pericarditis (n = 22), the relatively uncommon condition Kounis syndrome (n = 3) and, lastly, non-specific inflammation (n = 6).

Table 2 provides a summary of the cardiovascular complications categorized based on dose and brand information of the COVID-19 vaccines. The majority of cardiac complications occurred following the second dose of the COVID-19 vaccine (69.1%), followed by the first dose (25.1%), with the notable exception of acute myocardial infarction (AMI) where the majority of cases follow the first vaccine dose and mostly on the first day of vaccination. Regarding the brand of COVID-19 vaccine, Pfizer COVID-19 vaccines reported the highest prevalence (62.2%) in terms of cardiac complications, followed by the Moderna COVID-19 vaccine (25.5%), with other brands constituting a comparatively lower percentage. Thus, mRNA vaccines (Pfizer and Moderna) accounted for the majority (87.7%) of cardiac complications. However, it is interesting to note that this does not hold true for AMI, for which 60% of cases were associated with the vector-based vaccine AstraZeneca.

The spectrum of neurological complications and their outcomes are listed in Table 3. Most cases (n = 414, 94.3%) were non-fatal and 53.1% (n = 233) involved the central nervous system. The most prevalent complications were polyneuropathy (n = 146, 33.3%), demyelinating disorders (n = 109, 24.8%) and cerebrovascular disorders (n = 65, 14.8%). Less common were cranial nerve palsies (n = 32, 7.3%), plexopathy (n = 23, 5.2%), inflammatory disorders (n = 22, 5.0%) and neuro-immunological disorders (n = 18, 4.1%). Based on specific diagnoses, the most prevalent complication was GBS (n = 139, 31.7%). This was followed by ischemic stroke (n = 58, 13.2%), multiple sclerosis (n = 41 or 9.3%), acute demyelinating encephalomyelitis (ADEM) (n = 26, 5.9%), acute transverse myelitis (n = 21, 4.8%), facial nerve palsy (n = 21, 4.8%) and other relatively uncommon disorders such as plexopathy/brachial neuritis (n = 17, 3.9%) and longitudinal extensive transverse myelitis (LETM) (n = 14, 3.2%). Table 4 presents a tabulation of the cases with neurological complications, categorized by dose and brand of the COVID-19 vaccine received. Overall, neurological complications were more common after the first dose of the COVID-19 vaccine (63.6%) than after the second dose (23.7%). However, this pattern did not hold true for plexopathy and other peripheral nervous system complications, as these complications were more commonly observed after the second dose than the first. With regard to the COVID-19 vaccine, AstraZeneca was reported as the brand most commonly (46.0%) associated with neurological complications, followed by Pfizer (30.8%) and Moderna (10.3%). Upon closer examination, the Pfizer vaccine was more commonly associated with central nervous system inflammatory disorders (50.0%), followed by AstraZeneca (31.8%) and Moderna (13.6%). Another notable observation regarding the Pfizer vaccine is that it was the primary vaccine associated with cranial nerve disorders (53.1%) and plexopathy (47.8%).

## 4. Discussion

### 4.1. Cardiac Complications

Cardiac complications have been associated with both COVID-19 infections and COVID-19 vaccinations. Zuin M et al. (2022) estimated the occurrence of myocarditis to be 0.21 per 1000 COVID-19 patients compared to 0.09 per 1000 control subjects who were not infected but developed myocarditis within the same time frame, thus giving a hazard ratio of 5.15 within one year of the index infection [386], highlighting the significantly increased risk of myocarditis with infection.

Voleti N et al. (2022) compared the occurrence of myocarditis between 55.5 million COVID-19-vaccinated individuals and 2.5 million COVID-19 patients and found the odds of developing myocarditis to be higher in patients with COVID-19 (relative risk = 14.8, 95% CI = 11.1, 19.8) compared to COVID-19-vaccinated individuals (relative risk = 2.0, 95% CI = 1.4, 2.7) [387]. Hence, the risk of myocarditis is much higher in individuals who are infected with SARS-CoV2 that in those who received the COVID-19 vaccine, underscoring the importance of vaccination against the virus.

Ling RR et al. (2022) found no significant difference in the incidence of myopericarditis between those who were vaccinated against SARS-CoV2 (18.2 cases [10.9–30.3] per million doses, high certainty) and the general population (56.0 [10.7–293.7], moderate certainty, *p* = 0.20) [388]. These observations by Ling RR et al. suggest that COVID-19 vaccination does not increase a person’s risk of myopericarditis specifically.

Based on the above, it would appear that the risk of cardiac complications following COVID-19 far outweighs that following COVID-19 vaccination, thereby supporting the importance of vaccination to reduce the risk of infection, particularly in high-risk people.

### 4.2. Mechanisms Underlying Cardiac Complications following COVID-19 Vaccination

Different hypotheses have been proposed for the occurrence of cardiac complications following COVID-19 vaccination. One of these is the molecular mimicry hypothesis, which states that antibodies mounted against the vaccine cross-react with cardiac antigens [389,390,391]. This proposed mechanism has been both supported [12] and refuted [13] by research findings. An alternative theory is that the host response directed against the mRNA component of vaccines causes the activation and dysregulation of immunological responses, leading to the development of myocarditis as part of a systemic reaction [392,393]. In both cases, these aberrant responses are more likely to involve individuals with underlying genetic predispositions. It is pertinent to note that different complications could have different underlying pathogeneses in relation to the vaccine, an area that requires further investigation.

### 4.3. Neurological Complications

Neurological complications following COVID-19 vaccinations are mostly mild and transient. The more serious illnesses present as strokes, meningitis encephalitis, neuromyelitis optica (NMO), acute disseminated encephalomyelitis (ADEM), a relapse of multiple sclerosis (MS), transverse myelitis (TM) and peripheral neuropathy (GBS and CIDP). Similar complications also occur with COVID-19, but generally at a much higher frequency. In this review, the most frequently reported post-vaccination disorders were GBS (n = 139, 31.7%), ischemic stroke (n = 58, 13.2%) and a group of demyelinating disorders comprising multiple sclerosis, (n = 41, 9.3%), transverse myelitis (n = 35, 8.0%) and ADEM (n = 26, 5.9%).

### 4.4. Neurological Complications in COVID-19-Vaccinated People Compared to COVID-19 Patients and/or Uninfected People

#### 4.4.1. Guillain–Barré Syndrome (GBS)

GBS, an uncommon immune-mediated acute polyneuropathy that is often triggered by preceding viral or bacterial infections, has a worldwide distribution with a pooled global incidence estimated to be 0.81 to 1.89 per 100,000 per year [394]. COVID-19 is associated with an increased risk of GBS, with an adjusted odds ratio (AOR) of 3.27 (95% CI = 1.32, 8.09) compared to non-infected controls [395]. The pooled incidence of GBS among COVID-19 vaccine recipients, estimated to be 8.1 per 1,000,000 vaccinations [396], is also noted to be significantly higher than the reported annual incidence in the general population.

The classic form of GBS, a demyelinating neuropathy with rapidly ascending weakness, is the most common subtype [397,398]. An axonal form is also quite common, especially in Central and South America and the Far East [399,400,401]. Other well-recognised forms are Miller Fisher syndrome (MFS) and the facial diplegia subtypes. In this review, classic GBS is indeed the most common form, constituting about 84% of all cases, mirroring the worldwide distribution. Other subtypes reported include the axonal form (8.2%), MFS (4.8%) and facial diplegia (2.7%). Their age and gender distributions also appear to be similar to those widely reported, with a mean age of 55 years and a male preponderance of 61.6%. In terms of vaccine association, most (61%) cases reported taking viral vector vaccines, a finding that is similar to that of many other reports [153,402].

#### 4.4.2. Cerebrovascular Disorders

Stroke ranks as the second leading cause of death globally, accounting for 11% of all deaths [403]. Ischaemic strokes made up 62.4% of all incident strokes in 2019 [404]. In COVID-19 patients, the pooled prevalence of ischemic stroke was estimated to be 2% (95% CI 1–2%; *p* < 0.01) [405] and the risk of ischaemic stroke is significantly higher than that in non-COVID-19 patients (pooled relative risk: 2.41; 95% CI 1.08–5.38) [406]. In a meta-analysis by Stefanou M-I et al. (2022), the pooled proportion of acute ischemic stroke following exposure to any type of COVID-19 vaccine was 4.7 cases per 100,000 vaccinations (95% CI 2.2–8.1; I^2^ = 99.9%) [407]. The relative risks following COVID-19 and COVID-19 vaccination have not been compared to date.

In this review, ischaemic stroke is the second most prevalent complication following COVID-19 vaccination. It accounts for about 90% of all stroke cases and has a fatality rate of 27.6%. As reported by other studies [408,409], it is more common in females (60.7%) and mostly (78.1%) associated with viral vector vaccines. Its causes are cerebral arterial occlusion and venous occlusion or cerebral venous thrombosis (CVT). Contrary to expectations, arterial occlusion only accounted for 41.1% of cases, while CVT, generally considered to be very rare, accounted for 58.9%. The annual incidence of CVT has been reported by others to be 2–15.7/10,000,000 [410].

The incidence of CVT in SARS-CoV-2 infections was found to be 8.8 per 10,000 with a male predominance and average age of 49 years [411]. In the TriNetX COVID-19 Research Network platform (www.trinetx.com), which is used to compare the prevalence of CVT in the setting of COVID-19 patients versus the uninfected population [412], the odds ratio for CVT in COVID-19 patients was 40.99 [95% CI = 30.11–55.81]. Further, a retrospective cohort study found the incidence of CVT in the two weeks after a COVID-19 diagnosis to be 42.8 per million people (95% CI 28.5–64.2), which was significantly higher than in a matched cohort of people who received an mRNA vaccine (RR = 6.33, 95% CI 1.87–21 [413]. Thus, the risk of CVT appears to be increased by a COVID-19 infection compared to those uninfected, and moderately increased compared to the COVID-19-vaccinated. Therefore, on balance, it would be more advantageous to be vaccinated despite the known risk of CVT post vaccination, for the simple reason that the risk with the infection is much higher. The risk can further be mitigated by avoiding a specific vaccine type, considering that the great majority (n = 54 out of 65, 83.1%) of cases are associated with vector-based vaccines (Refer to Table 3).

Other neurological events encountered in our study are relatively uncommon and include the reactivation/relapse of multiple sclerosis (9.4%), acute demyelinating encephalomyelitis (ADEM) (5.9%), acute transverse myelitis and facial nerve palsy (4.8% in both instances), plexopathy/brachial neuritis (3.9%) and longitudinal extensive transverse myelitis (LETM) (3.2%), all of which are also associated with COVID-19.

### 4.5. Mechanisms Underlying Neurological Complications following COVID-19 Vac-cination

The mechanism by which a COVID-19 vaccination could trigger neurological complications is generally attributed to molecular mimicry in the background of genetic susceptibility and pre-existing comorbidities. In the case of strokes, traditional risk factors such as hypertension, diabetes and hyperlipidaemia are likely to act in concert with the hypercoagulability seen in COVID-19 and after COVID-19 vaccination. Further research is needed to elucidate the pathways involved.

### 4.6. Limitations

This systematic review utilizes case series and case reports as its data sources. Although case series and case reports generally provide lower-quality evidence compared to cross-sectional studies, they provide in-depth descriptions of individual cases, which are essential for the investigation of vaccination–complication associations. Another limitation of note is the tendency for only unusual or exceptional cases to be published. This selective reporting can lead to an underestimation of the true prevalence and distribution of post-vaccination complications in the population, as more common or less striking cases may go unreported. Language bias is another possible limitation, given that this review exclusively considered case reports published in English through specific databases, inevitably overlooking cases published in other languages or in less widely recognized journals. Regional bias is also a concern, as cases reported in specific regions or countries may be more accessible, resulting in a skewed geographical representation of the reported cases. Additionally, diagnostic bias is a potential source of concern, as cases with clear and easily identifiable complications may be more likely to be reported compared to cases with more complex complications, introducing a bias toward certain types of complications. Furthermore, it is acknowledged that adverse events are expected when a large population is vaccinated, as in the case of the COVID-19 pandemic, thereby precluding a meaningful statistical analysis. This is particularly so in the absence of control groups for comparisons. Lastly, this review is based on publications collected over a relatively short time period, and quite soon after the initiation of the vaccination program, which necessarily limits the observed complications to those which are more acute in nature.

## 5. Conclusions

This systematic review highlights the potential of cardiac and neurological complications following COVID-19 vaccination, as reported through case reports and case series. The most frequently reported complications were myocarditis, Guillain–Barré syndrome (GBS), ischemic stroke and exacerbations of multiple sclerosis. However, it is essential to interpret these findings within the broader context of the available evidence.

It is crucial to acknowledge that the true prevalence and distribution of post-vaccination complications in the general population may be underestimated due to potential biases in case reporting and publication. Additionally, the mechanisms underlying these complications are not fully elucidated, and further research is needed to understand the pathways involved.

Continuous surveillance, the close monitoring of vaccinated individuals and the prompt treatment of relevant complications are essential, especially for those with associated risk factors or pre-existing comorbidities. Healthcare providers should remain vigilant and provide appropriate counselling to patients regarding the potential risks and benefits of COVID-19 vaccination.

## Figures and Tables

**Table 1:** Summary of cases with cardiovascular complications post COVID-19 vaccination (n = 259).

Complications	Total Cases, n (%)	Demography, n (%)			Geographical Area, n (%)					Vaccine Type, n (%)			Clinical Course/Outcomes n (%)		
		Age, Mean ± SD 33.8 ± 19.1	F 120 (46.3)	M 139 (53.7)	Asia 100 (38.6)	Africa 1 (0.4)	America 107 (41.3)	Europe 49 (18.9)	Australasia 2 (0.8)	mRNA 233 (90.0) *	Viral vector 22 (8.5)	Attenuated virus 2 (1.5)	† 214 (82.6)	†† 27 (10.9)	Fatal 6 (2.4)
Cardiac arrhythmia	3 (1.2)	44.7 ± 13.1	1 (33.3)	2 (66.7)	2 (66.7)	0 (0.0)	1 (33.3)	0 (0.0)	0 (0.0)	2 (66.7)	0 (0.0)	1 (33.3)	2 (66.7)	1 (33.3)	0 (0.0)
Cardiomyopathy	11 (4.2)	54.5 ± 19.3	10 (90.9)	1 (9.1)	3 (27.3)	0 (0.0)	3 (27.3)	4 (36.4)	1 (9.1)	9 (81.8)	2 (18.2)	0 (0.0)	11 (100.0)	0 (0.0)	0 (0.0)
Inflammatory conditions of the heart	221 (85.3)	30.8 ± 16.5	93 (42.1)	128 (57.9)	75 (33.9)	1 (0.5)	100 (45.0)	44 (19.9)	1 (0.5)	207 (93.7)	13 (5.9)	1 (0.5)	181 (85.4)	24 (11.4)	6 (2.8)
Kounis syndrome Type 1	2	42.3 ± 21.0	2	0	2	0	0	0	0	1	0	1	2	0	0
Kounis syndrome Type 3	1	NA	0	1	1	0	0	0	0	1	0	0	1	0	0
Myocarditis	152	28.0 ± 13.8	49	103	64	1	59	27	1	142	10	0	121	16	5
Myopericarditis	38	19.7 ± 8.4	27	11	4	0	31	3	0	37	1	0	34	4	0
Pericarditis	22	54.1 ± 20.5	14	8	4	0	5	13	0	20	2	0	17	4	1
Non-specific inflammation	6	25.3 ± 6.8	1	5	0	0	5	1	0	6	0	0	6	0	0
Acute myocardial infarction	15 (5.8)	66.0 ± 18.0	8 (53.3)	7 (46.7)	12 (80.0)	0 (0.0)	3 (20.0)	0 (0.3)	0 (0.0)	6 (40.0)	7 (46.7)	2 (13.3)	12 (85.7)	2 (14.3)	0 (0.0)
Myocardial injury	9 (3.5)	24.8 ± 18.4	8 (88.9)	1 (11.1)	8 (100.0)	0 (0.0)	0 (0.0)	0 (0.0)	0 (0.0)	8 (100.0)	0 (100.0)	0 (100.0)	8 (100.0)	0 (0.0)	0 (0.0)
Occlusion of coronary artery	2	54.0 ± 22.6	1	1	1	0	0	1	0	2	0	0	1	0	0
Transient injury	7	16.4 ± 0.5	7	0	7	0	0	0	0	7	0	0	7	0	0

Note: † uncomplicated cases; †† complicated cases. * The total number of mRNA vaccines (n = 233) includes Pfizer (n = 161), Moderna (n = 66), mRNA vaccine, brands not specified (n = 4) and 2 cases under “heterologous vaccines”, a combination of AstraZeneca (1st dose) and Pfizer (2nd dose), where the cardiac complication occurred following the second dose.

**Table 2:** Summary of cases with cardiovascular complications, categorized by dose and brand information for COVID-19 vaccines (n = 259).

Complications	Total Cases, n (%)	Dose of Vaccine, n (%)				Brand of Vaccine, n (%)								
		First 65 (25.1)	Second 179 (69.1)	First Booster 6 (2.3)	NA 9 (3.5)	Astrazeneca 17 (6.6)	Jassen 4 (1.5)	Moderna 66 (25.5)	Pfizer 161 (62.2)	Sinopharm 1 (0.4)	Sinovac 3 (1.2)	Sputnik V 1 (0.4)	Nonspecific mRNA 4 (1.5)	Heterologous 2 (0.8)
Cardiac arrhythmia	3 (1.2)	1 (33.3)	1 (33.3)	1 (33.3)	0 (0.0)	0 (0.0)	0 (0.0)	0 (0.0)	2 (66.7	1 (33.3)	0 (0.0)	0 (0.0)	0 (0.0)	0 (0.0)
Cardiomyopathy	11 (4.2)	6 (54.5)	5 (45.5)	0 (0.0)	0 (0.0)	2 (18.2)	0 (0.0)	4 (36.4)	5 (45.5)	0 (0.0)	0 (0.0)	0 (0.0)	0 (0.0)	0 (0.0)
Inflammatory conditions of the heart	221 (85.3)	45 (20.4)	166 (75.1)	5 (2.3)	5 (2.3)	8 (3.6)	4 (1.8)	58 (26.2)	143 (64.7)	0 (0.0)	1 (0.5)	1 (0.5)	4 (1.8)	2 (0.9)
Kounis syndrome Type 1	2	2	0	0	0	0	0	0	1	0	1	0	0	0
Kounis syndrome Type 3	1	1	0	0	0	0	0	0	1	0	0	0	0	0
Myocarditis	152	29	114	5	4	5	4	48	90	0	0	1	2	2
Myopericarditis	38	2	36	0	0	1	0	6	31	0	0	0	0	0
Pericarditis	22	9	12	0	1	2	0	3	15	0	0	0	2	0
Non-specific inflammation	6	2	4	0	0	0	0	1	5	0	0	0	0	0
Acute myocardial infarction	15 (5.8)	10 (66.7)	1 (6.7)	0 (0.0)	4 (26.7)	7 (46.7)	0 (0.0)	4 (26.7)	2 (13.3)	0 (0.0)	2 (13.3)	0 (0.0)	0 (0.0)	0 (0.0)
Myocardial injury	9 (3.5)	3 (33.3)	6 (66.7)	0 (0.0)	0 (0.0)	0 (0.0)	0 (0.0)	0 (0.0)	9 (100.0)	0 (0.0)	0 (0.0)	0 (0.0)	0 (0.0)	0 (0.0)
Occlusion of coronary artery	2	1	1	0	0	0	0	0	2	0	0	0	0	0
Transient injury	7	2	5	0	0	0	0	0	7	0	0	0	0	0

**Table 3:** Summary of cases with neurological complications post COVID-19 vaccination (n = 439).

Complications	Total Cases, n (%)	Demography, n (%)			Geographical Area, n (%)					Vaccine Type, n (%)			Clinical Course/Outcomes, n (%)	
		Age, Mean ± SD 49.7 ± 17.0	F 230 (53.5)	M 200 (46.5)	Asia 195 (44.4)	Africa 2 (0.5)	America 99 (22.6)	Europe 130 (29.6)	Australasia 13 (3.0)	mRNA 183 (42.1)	Viral Vector 233 (53.6) *	Attenuated Virus 19 (4.4)	Non-Fatal 414 (94.3)	Fatal 25 (5.7)
CNS—Cerebrovascular disorders	65 (14.8)	47.5 ± 15.5	41 (73.2)	15 (26.8)	22 (33.8)	0 (0.0)	8 (12.3)	34 (52.3)	1 (1.5)	10 (15.6)	54 (84.4)	0 (0.0)	48 (73.8)	17 (26.2)
Haemorrhagic stroke	7	57.4 ± 13.7	7	0	4	0	1	2	0	3	4	0	6	1
Ischaemic stroke	58	46.0 ± 15.4	34	15	18	0	7	32	1	7	50	0	42	16
CNS—Demyelinating disorders	109 (24.8)	43.6 ± 16.5	74 (67.9)	35 (32.1)	51 (46.8)	0 (0.0)	24 (22.0)	32 (29.4)	2 (1.8)	51 (47.2)	50 (46.3)	7 (6.5)	105 (96.3)	4 (3.7)
ADEM	26	46.2 ± 17.4	15	11	19	0	1	4	2	5	17	4	24	1
ADEM (AHLE)	4	42.3 ± 18.4	3	1	1	0	0	3	0	0	4	0	3	1
ATM	21	45.3 ± 20.9	13	8	15	0	4	2	0	11	8	2	21	0
LETM	14	44.9 ± 16.1	7	7	10	0	3	1	0	2	11	1	14	0
Multiple sclerosis	41	41.0 ± 13.6	33	8	3	0	16	22	0	33	7	0	40	1
Others	3	40.3 ± 16.6	3	0	3	0	0	0	0	0	3	0	3	0
CNS—Inflammatory disorders	22 (5.0)	47.9 ± 18.3	15 (68.2)	7 (31.8)	13 (59.1)	0 (0.0)	3 (13.6)	6 (27.3)	0 (0.0)	15 (68.2)	7 (31.8)	0 (0.0)	22 (100.0)	0 (0.0)
Encephalitis	11	52.1 ± 18.1	6	5	6	0	1	4	0	6	5	0	11	0
Meningitis	9	42.3 ± 16.8	7	2	6	0	1	2	0	7	2	0	9	0
Meningoencephalitis	2	49.5 ± 31.8	2	0	1	0	1	0	0	2	0	0	2	0
CNS—Neuroimmunological disorders	18 (4.1)	43.2 ± 13.5	10 (55.6)	8 (44.4)	13 (72.2)	0 (0.0)	4 (22.2)	1 (5.6)	0 (0.0)	5 (27.8)	11 (61.1)	2 (11.1)	18 (100.0)	0 (0.0)
MOGOM	4	41.8 ± 10.5	2	2	4	0	0	0	0	0	4	0	4	0
NMOSD	9	49.4 ± 15.4	7	2	5	0	3	1	0	4	3	2	9	0
Optic neuritis	5	34.4 ± 7.8	1	4	4	0	1	0	0	1	4	0	5	0
CNS—Others	19 (4.3)	56.1 ± 20.4	10 (52.6)	9 (47.4)	10 (52.6)	0 (0.0)	5 (26.3)	3 (15.8)	1 (5.3)	10 (52.6)	9 (47.4)	0 (0.0)	17 (89.5)	2 (10.5)
Cognitive deficit	1	NA	0	1	1	0	0	0	0	0	1	0	1	0
Delirium	1	NA	0	1	0	0	1	0	0	1	0	0	1	0
Dementia	1	NA	1	0	1	0	0	0	0	0	1	0	0	1
Encephalopathy	7	65.4 ± 18.6	4	3	3	0	3	0	1	4	3	0	6	1
FND	2	37.0 ± 1.4	2	0	0	0	0	2	0	2	0	0	2	0
Migraine	1	NA	1	0	0	0	0	1	0	1	0	0	1	0
OMAS	1	NA	0	1	1	0	0	0	0	0	1	0	1	0
Seizures	5	43.6 ± 20.4	2	3	4	0	1	0	0	2	3	0	5	0
MIX CNS/PNS	2 (0.5)	51.5 ± 6.4	0 (0.0)	2 (100.0)	1 (50.0)	0 (0.0)	0 (0.0)	1 (50.0)	0 (0.0)	0 (0.0)	2 (100.0)	0 (0.0)	2 (100.0)	0 (0.0)
PNS—Polyneuropathy	146 (33.3)	55.1 ± 17.0	56 (38.4)	90 (61.6)	65 (44.5)	2 (1.4)	35 (24.0)	35 (24.0)	9 (6.2)	49 (33.6)	89 (61.0)	8 (5.4)	144 (98.6)	2 (1.4)
CIDP	7	59.0 ± 14.9	2	5	1	0	2	3	1	3	4	0	7	0
GBS	139	54.9 ± 17.2	54	85	64	2	33	32	8	46	85	8	139	0
PNS—Cranial nerve disorders	32 (7.3)	49.6 ± 14.9	16 (50.0)	16 (50.0)	14 (43.8)	0 (0.0)	6 (18.8)	12 (37.5)	0 (0.0)	23 (74.2)	6 (19.4)	2 (6.5)	32 (100.0)	0 (0.0)
Facial nerve palsy	21	45.2 ± 12.7	10	11	10	0	4	7	0	12	6	2	21	0
Others	11	58.0 ± 15.9	6	5	4	0	2	5	0	11	0	0	11	0
PNS—Plexopathy	23 (5.2)	51.8 ± 13.4	6 (26.1)	17 (73.9)	5 (21.7)	0 (0.0)	13 (56.5)	5 (21.7)	0 (0.0)	17 (77.3)	5 (22.7)	0 (0.0)	23 (100.0)	0 (0.0)
Brachial neuritis	17	51.5 ± 11.6	4	13	3	0	9	5	0	13	3	0	17	0
Brachial plexus neuropraxia	5	54.4 ± 20.7	1	4	1	0	4	0	0	4	1	0	5	0
Lumbosacral plexus neuropathy	1	NA	1	0	1	0	0	0	0	0	1	0	1	0
PNS—Others	3 (0.7)	47.7 ± 20.6	2 (66.7)	1 (33.3)	1 (33.3)	0 (0.0)	1 (33.3)	1 (33.3)	0 (0.0)	3 (100.0)	0 (0.0)	0 (0.0)	3 (100.0)	0 (0.0)
Cubital tunnel syndrome	1	NA	0	1	0	0	0	1	0	1	0	0	1	0
Monoplegia	1	NA	1	0	1	0	0	0	0	1	0	0	1	0
Myasthenia Gravis	1	NA	1	0	0	0	1	0	0	1	0	0	1	0

Note: * The total number of viral vector vaccine recipients was 233, comprising homologous AstraZeneca (n = 202), Janssen (n = 16), Sputnik V (n = 13) and viral vector vaccines with the brand not specified (n = 2). Acute demyelinating encephalomyelitis (ADEM). Acute haemorrhagic leukoencephalitis (AHLE). Chronic Inflammatory Demyelinating Polyneuropathy (CIPD). Central nervous system (CNS). Functional neurological disorders (FND). Guillain–Barré syndrome (GBS). Longitudinal extensive transverse myelitis (LETM). MOG-associated opticomyelopathy (MOGOM). Neuromyelitis Optica Spectrum Disorder (NMOSD). Opsoclonus myoclonus ataxia syndrome (OMAS). Peripheral nervous system (PNS).

**Table 4:** Summary of cases with neurological complications, categorized by dose and brand information for COVID-19 vaccines (n = 439).

Complications	Total Cases, n (%)	Dose of Vaccine n (%)				Brand of Vaccine n (%)									
		First 279 (63.6)	Second 104 (23.7)	First Booster 1 (0.2)	NA 55 (12.5)	Astrazeneca 202 (46.0)	Jassen 16 (3.6)	Moderna 45 (10.3)	Pfizer 135 (30.8)	Sinopharm 9 (2.1)	Sinovac 6 (1.4)	Sputnik V 13 (3.0)	Nonspecific mRNA 9 (2.1)	Heterologous 1 (0.2)	Covaxin Bharat 3 (0.7)
CNS—Cerebrovascular disorders	65 (14.8)	39 (60.0)	4 (6.2)	1 (1.5)	21 (32.3)	49 (75.4)	5 (7.7)	1 (1.5)	9 (13.8)	0 (0.0)	0 0.0)	0 (0.0)	1 (1.7)	0 (0.0)	0 (0.0)
Haemorrhagic stroke	7	6	1	0	0	4	0	0	3	0	0	0	0	0	0
Ischaemic stroke	58	33	3	1	21	45	5	1	6	0	0	0	1	0	0
CNS—Demyelinating disorders	109 (24.8)	69 (63.3)	35 (32.1)	0 (0.0)	5 (4.6)	47 (43.1)	1 (0.9)	15 (13.8)	36 (33.0)	3 (2.8)	2 (1.8)	2 (1.8)	1 (0.9)	0 (0.0)	2 (1.8)
ADEM	26	18	7	0	1	16	0	2	3	2	1	1	0	0	1
ADEM (AHLE)	4	4	0	0	0	4	0	0	0	0	0	0	0	0	0
ATM	21	15	5	0	1	8	0	4	7	0	1	0	0	0	1
LETM	14	12	2	0	0	11	0	0	2	1	0	0	0	0	0
MS	41	18	20	0	3	5	1	9	24	0	0	1	1	0	0
Others	3	2	1	0	0	3	0	0	0	0	0	0	0	0	0
CNS—Inflammatory disorders	22 (5.0)	10 (45.5)	9 (40.9)	0 (0.0)	3 (13.6)	7 (3.18)	0 (0.0)	3 (13.6)	11 (50.0)	0 (0.0)	0 (0.0)	0 (0.0)	0 (0.0)	1 (4.5)	0 (0.0)
Encephalitis	11	3	5	0	1	5	0	3	2	0	0	0	0	1	0
Meningitis	9	5	4	0	0	2	0	0	7	0	0	0	0	0	0
Meningoencephalitis	2	2	0	0	0	0	0	0	2	0	0	0	0	0	0
CNS—Neuroimmunological disorders	18 (4.1)	15 (83.3)	3 (16.7)	0 (0.0)	0 (0.0)	11 (61.1)	0 (0.0)	1 (5.6)	4 (22.2)	0 (0.0)	1 (5.6)	0 (0.0)	1 (5.6)	0 (0.0)	0 (0.0)
MOGON	4	4	0	0	0	4	0	0	0	0	0	0	0	0	0
NMOSD	9	7	2	0	0	3	0	1	3	0	1	0	1	0	0
Optic neuritis	5	4	1	0	0	4	0	0	1	0	0	0	0	0	0
CNS—Others	19 (4.3)	14 (73.7)	5 (26.3)	0 (0.0)	0 (0.0)	9 (47.4)	0 (0.0)	4 (21.1)	6 (31.6)	0 (0.0)	0 (0.0)	0 (0.0)	0 (0.0)	0 (0.0)	0 (0.0)
Cognitive deficit	1	1	0	0	0	1	0	0	0	0	0	0	0	0	0
Delirium	1	1	0	0	0	0	0	0	1	0	0	0	0	0	0
Dementia	1	0	1	0	0	1	0	0	0	0	0	0	0	0	0
Encephalopathy	7	6	1	0	0	3	0	3	1	0	0	0	0	0	0
FND	2	1	1	0	0	0	0	1	1	0	0	0	0	0	0
Migraine	1	0	1	0	0	0	0	0	1	0	0	0	0	0	0
OMAS	1	0	1	0	0	1	0	0	0	0	0	0	0	0	0
Seizures	5	5	0	0	0	3	0	0	2	0	0	0	0	0	0
MIX CNS/PNS	2 (0.4)	1 (50.0)	0 (0.0)	0 (0.0)	1 (50.0)	1 (50.0)	1 (50.0)	0 (0.0)	0 (0.0)	0 (0.0)	0 (0.0)	0 (0.0)	0 (0.0)	0 (0.0)	0 (0.0)
PNS—Polyneuropathy	146 (33.3)	105 (71.9)	25 (17.1)	0 (0.0)	16 (11.0)	70 (47.9)	8 (5.5)	7 (4.8)	41 (28.1)	6 (4.1)	2 (1.4)	9 (6.2)	3 (2.1)	0 (0.0)	0 (0.0)
PNS—Cranial nerve disorders	32 (7.3)	18 (56.3)	9 (28.1)	0 (0.0)	5 (15.6)	3 (9.4)	1 (3.1)	6 (18.8)	17 (53.1)	0 (0.0)	1 (3.1)	2 (6.3)	1 (3.1)	0 (0.0)	1 (3.1)
Facial nerve palsy	21	11	6	0	4	3	1	5	7	0	1	2	1	0	1
Others	11	7	3	0	1	0	0	1	10	0	0	0	0	0	0
PNS—Plexopathy	23 (5.2)	7 (30.4)	12 (52.2)	0 (0.0)	4 (17.4)	5 (21.7)	0 (0.0)	6 (26.1)	11 (47.8)	0 (0.0)	0 (0.0)	0 (0.0)	1 (4.3)	0 (0.0)	0 (0.0)
Brachial neuritis	17	6	9	0	2	3	0	5	8	0	0	0	1	0	0
Brachial plexus neuropraxia	5	1	3	0	1	1	0	1	3	0	0	0	0	0	0
Lumbosacral plexus neuropathy	1	0	0	0	1	1	0	0	0	0	0	0	0	0	0
PNS—Others	3 (0.7)	1 (33.3)	2 (66.7)	0 (0.0)	0 (0.0)	0 (0.0)	0 (0.0)	2 (66.7)	0 (0.0)	0 (0.0)	0 (0.0)	0 (0.0)	1 (33.3)	0 (0.0)	0 (0.0)
Cubital tunnel syndrome	1	1	0	0	0	0	0	1	0	0	0	0	0	0	0
Monoplegia	1	0	1	0	0	0	0	0	0	0	0	0	1	0	0
Myasthenia Gravis	1	0	1	0	0	0	0	1	0	0	0	0	0	0	0

## Data Availability

Not applicable.

## Supplementary Materials

The following supporting information can be downloaded at https://www.mdpi.com/article/10.3390/vaccines12060575/s1, Supplementary S1: PRISMA checklist. Supplementary S2: Search strategies (searches were performed on 14 February 2022). Supplementary S3: Detailed description of studies reporting cases with cardiac complications following COVID-19 vaccination (n = 134). Supplementary S4: Detailed description of studies reporting cases with neurological complications following COVID-19 vaccination (n = 245).
