# Supplementary material for: Cardiac and Neurological Complications Post COVID-19 Vaccination: A Systematic Review of Case Reports and Case Series
Source: Vaccines (Basel). 2024 May 24;12(6):575. doi: 10.3390/vaccines12060575 (PMC11209191; doi:10.3390/vaccines12060575)
Supplement: Supplementary file 1 [file vaccines-12-00575-s001.zip › vaccines-2965726-supplementary.pdf]

Supplementary S1: PRISMA checklist

Section and Topic	Item #	Checklist item	Location where item is reported
**TITLE**			
Title	1	Identify the report as a systematic review.	1
**ABSTRACT**			
Abstract	2	See the PRISMA 2020 for Abstracts checklist.	1
**INTRODUCTION**			
Rationale	3	Describe the rationale for the review in the context of existing knowledge.	1
Objectives	4	Provide an explicit statement of the objective(s) or question(s) the review addresses.	1
**METHODS**			
Eligibility criteria	5	Specify the inclusion and exclusion criteria for the review and how studies were grouped for the syntheses.	2
Information sources	6	Specify all databases, registers, websites, organisations, reference lists and other sources searched or consulted to identify studies. Specify the date when each source was last searched or consulted.	2
Search strategy	7	Present the full search strategies for all databases, registers and websites, including any filters and limits used.	2
Selection process	8	Specify the methods used to decide whether a study met the inclusion criteria of the review, including how many reviewers screened each record and each report retrieved, whether they worked independently, and if applicable, details of automation tools used in the process.	2
Data collection process	9	Specify the methods used to collect data from reports, including how many reviewers collected data from each report, whether they worked independently, any processes for obtaining or confirming data from study investigators, and if applicable, details of automation tools used in the process.	2
Data items	10a	List and define all outcomes for which data were sought. Specify whether all results that were compatible with each outcome domain in each study were sought (e.g. for all measures, time points, analyses), and if not, the methods used to decide which results to collect.	2
	10b	List and define all other variables for which data were sought (e.g. participant and intervention characteristics, funding sources). Describe any assumptions made about any missing or unclear information.	2
Study risk of bias assessment	11	Specify the methods used to assess risk of bias in the included studies, including details of the tool(s) used, how many reviewers assessed each study and whether they worked independently, and if applicable, details of automation tools used in the process.	N/A
Effect measures	12	Specify for each outcome the effect measure(s) (e.g. risk ratio, mean difference) used in the synthesis or presentation of results.	3
Synthesis methods	13a	Describe the processes used to decide which studies were eligible for each synthesis (e.g. tabulating the study intervention characteristics and comparing against the planned groups for each synthesis (item #5)).	3
	13b	Describe any methods required to prepare the data for presentation or synthesis, such as handling of missing summary statistics, or data conversions.	3
	13c	Describe any methods used to tabulate or visually display results of individual studies and syntheses.	3
	13d	Describe any methods used to synthesize results and provide a rationale for the choice(s). If meta-analysis was performed, describe the model(s), method(s) to identify the presence and extent of statistical heterogeneity, and software package(s) used.	3

Section and Topic	Item #	Checklist item	Location where item is reported
	13e	Describe any methods used to explore possible causes of heterogeneity among study results (e.g. subgroup analysis, meta-regression).	N/A
	13f	Describe any sensitivity analyses conducted to assess robustness of the synthesized results.	N/A
Reporting bias assessment	14	Describe any methods used to assess risk of bias due to missing results in a synthesis (arising from reporting biases).	N/A
Certainty assessment	15	Describe any methods used to assess certainty (or confidence) in the body of evidence for an outcome.	N/A
**RESULTS**			
Study selection	16a	Describe the results of the search and selection process, from the number of records identified in the search to the number of studies included in the review, ideally using a flow diagram.	3
	16b	Cite studies that might appear to meet the inclusion criteria, but which were excluded, and explain why they were excluded.	3
Study characteristics	17	Cite each included study and present its characteristics.	3
Risk of bias in studies	18	Present assessments of risk of bias for each included study.	N/A
Results of individual studies	19	For all outcomes, present, for each study: (a) summary statistics for each group (where appropriate) and (b) an effect estimate and its precision (e.g. confidence/credible interval), ideally using structured tables or plots.	Supplementary 1 and 2
Results of syntheses	20a	For each synthesis, briefly summarise the characteristics and risk of bias among contributing studies.	Table 1 and 2
	20b	Present results of all statistical syntheses conducted. If meta-analysis was done, present for each the summary estimate and its precision (e.g. confidence/credible interval) and measures of statistical heterogeneity. If comparing groups, describe the direction of the effect.	Table 3 and 4
	20c	Present results of all investigations of possible causes of heterogeneity among study results.	4
	20d	Present results of all sensitivity analyses conducted to assess the robustness of the synthesized results.	N/A
Reporting biases	21	Present assessments of risk of bias due to missing results (arising from reporting biases) for each synthesis assessed.	N/A
Certainty of evidence	22	Present assessments of certainty (or confidence) in the body of evidence for each outcome assessed.	N/A
**DISCUSSION**			
Discussion	23a	Provide a general interpretation of the results in the context of other evidence.	4-7
	23b	Discuss any limitations of the evidence included in the review.	7
	23c	Discuss any limitations of the review processes used.	7
	23d	Discuss implications of the results for practice, policy, and future research.	7
**OTHER INFORMATION**			

Section and Topic	Item #	Checklist item	Location where item is reported
Registration and protocol	24a	Provide registration information for the review, including register name and registration number, or state that the review was not registered.	7-8
	24b	Indicate where the review protocol can be accessed, or state that a protocol was not prepared.	7-8
	24c	Describe and explain any amendments to information provided at registration or in the protocol.	7-8
Support	25	Describe sources of financial or non-financial support for the review, and the role of the funders or sponsors in the review.	8
Competing interests	26	Declare any competing interests of review authors.	8
Availability of data, code and other materials	27	Report which of the following are publicly available and where they can be found: template data collection forms; data extracted from included studies; data used for all analyses; analytic code; any other materials used in the review.	8

Supplementary S2: Search strategies (searches were performed on 14 February 2022).				
		PubMed	Medline via Ebscohot	Scopus
1	COVID-19 vaccine	4903	16, 181	54, 705
2	Safety OR adverse event OR adverse reaction OR side-effect OR complication OR manifestation OR impairment OR disorder OR disease	5,024,891	6, 752, 246	1, 912, 149
3	Nervous system OR neurological	533,241	526, 892	2, 368, 095
4	Cardiovascular OR heart	1,289,867	1, 259, 936	6, 142, 793
5	review OR systematic review OR meta-analysis	1,983,732	2, 095, 855	33, 258, 453
6	(Nervous system OR neurological)<br>OR (Cardiovascular OR heart)	1,784,381	1, 748, 574	7, 989, 924
7	(COVID-19 vaccine) AND<br>(Safety OR adverse event OR adverse reaction OR side-effect OR complication OR manifestation OR impairment OR disorder OR disease)<br>AND<br>(Nervous system OR neurological)<br>OR (Cardiovascular OR heart)<br>NOT (review OR systematic review OR meta-analysis)	80	283	202

Supplementary S3: Detailed description of studies reporting cases with cardiac complications following COVID-19 vaccination (n = 134).

Author [reference]	Year	Country	Number of cases	Age range	Gender	Vaccines	Diagnosis	Outcome
Abbate et al., [1]	2021	USA	2	27-34	M (1); F (1)	Pfizer (2)	Pericarditis (1); Myopericarditis (1)	Discharged (1); Died (1)
Ahmed et al., [2]	2022	Iraq	7	16-36	M (7)	Pfizer (5); Moderna (2)	Myocarditis (7)	Discharged (7)
Aiba et al., [3]	2021	Japan	1	46	F (1)	Pfizer (1)	Premature ventricular contraction; Non-sustained ventricular tachycardia (1)	Not described (1)
Albert et al., [4]	2021	USA	1	24	M (1)	Moderna (1)	Myocarditis (1)	Discharged (1)
Alizadeh et al., [5]	2022	Germany	1	29	M (1)	Astrazeneca (1)	Myocarditis (1)	Discharged (1)
Allam et al., [6]	2022	Lebanon	1	64	M (1)	Pfizer (1)	Kounis syndrome (1)	Not described (1)
Al-Rasbi et al., [7]	2022	Oman	1	37	M (1)	Pfizer (1)	Myocarditis (1)	Discharged (1)
Ambati et al., [8]	2021	USA	2	16-17	M (2)	Pfizer (2)	Myopericarditis (2)	Discharged (2)
Ameratunga et al., [9]	2022	New Zealand	1	57	F (1)	Pfizer (1)	Myocarditis (1)	Died (1)
Ansari et al., [10]	2022	Germany	1	23	M (1)	Moderna (1)	Myocarditis (1)	Discharged (1)
Ashaari et al., [11]	2021	Ireland	1	66	M (1)	Pfizer (1)	Pericarditis (1)	Discharged (1)
Azdaki et al., [12]	2021	Iran	1	70	M (1)	Astrazeneca (1)	Myocaditis (1)	Not described (1)
Bae et al., [13]	2022	South Korea	1	38	F (1)	Moderna (1)	Myocarditis (1)	Discharged (1)
Bartlett et al., [14]	2021	USA	1	46	M (1)	Pfizer (1)	Myopericarditis (1)	Discharged (1)
Bengal et al., [15]	2022	Germany	2	20-23	M (2)	Moderna (2)	Myocarditis (2)	Discharged (2)
Beshai et al., [16]	2022	USA	1	45	M (1)	Moderna (1)	Takotsubo Cardiomyopathy	Discharged (1)
Boivin et al., [17]	2021	USA	1	96	F (1)	Moderna (1)	Myocardial infarction (1)	Discharged (1)
Boscolo et al., [18]	2021	Switzerland	1	63	F (1)	Moderna (1)	Takotsubo Cardiomyopathy (1)	Not described (1)
Cereda et al., [19]	2021	Italy	1	21	M (1)	Pfizer (1)	Myocarditis (1)	Discharged (1)
Ceylan et al., [20]	2022	Turkey	1	57	F (1)	Pfizer (1)	Myocarditis (1)	Discharged (1)
Chachar et al., [21]	2021	Bahrain	1	24	M (1)	Pfizer (1)	Myocarditis (1)	Discharged (1)
Chamling et al., [22]	2021	Germany	3	20-68	M (2); F (1)	Pfizer (2); Astrazeneca (1)	Myocarditis (3)	Not described (3)
Chatterjee et al.,[23]	2021	India	1	63	M (1)	Astrazeneca (1)	Myocardial infarction (1)	Discharged (1)
Chelala et al., [24]	2022	USA	5	16-19	M (5)	Pfizer (4); Moderna (1)	Myocarditis (5)	Discharged (5)
Chellapandian et al., [25]	2022	Qatar	1	22	M (1)	Moderna (1)	Myocarditis (1)	Discharged (1)
Choi et al., [26]	2021	South Korea	1	22	M (1)	Pfizer (1)	Myocarditis (1)	Died (1)
Crane et al., [27]	2021	Australia	1	72	M (1)	Astrazeneca (1)	Takotsubo Cardiomyopathy (1)	Discharged (1)
D'Angelo et al., [28]	2021	Italy	1	30	M (1)	Pfizer (1)	Myocarditis (1)	Discharged (1)
Dlewati et al., [29]	2022	USA	1	48	M (1)	Moderna (1)	Myocarditis (1)	Discharged (1)
Ehrlich et al., [30]	2021	Germany	1	40	M (1)	Pfizer (1)	Myocarditis (1)	Discharged (1)
Elheet et al., [31]	2022	Saudi Arabia	1	32	M (1)	Astrazeneca (1)	Myocardial infarction (1)	Discharged (1)
Fearon et al., [32]	2021	USA	1	73	F (1)	Moderna (1)	Takotsubo syndrome (1)	Discharged (1)
Federico et al., [33]	2022	Italy	1	32	M (1)	Moderna (1)	Pericarditis (1)	Discharged (1)
Freise et al., [34]	2022	Germany	8	13-56	M (5); F (3)	Pfizer (5); Moderna (3)	Myocarditis (4); undetermined of myocarditis or	Discharged (4); Not

							pericarditis (4)	described (4)
Gautam et al., [35]	2021	USA	1	66	M (1)	Pfizer (1)	Myocarditis (1)	Discharged (1)
Gill et al., [36]	2022	USA	1	44	M (1)	Moderna (1)	Myopericarditis (1)	Discharged (1)
Giublin et al., [37]	2022	Brazil	1	18	M (1)	Pfizer (1)	Myopericarditis (1)	Discharged (1)
Habedank et al., [38]	2022	Germany	1	60	M (1)	Moderna (1)	Myocarditis (1)	Discharged (1)
Habib et al., [39]	2021	Qatar	1	37	M (1)	Pfizer (1)	Myocarditis (1)	Discharged (1)
Hassanzadeh et al., [40]	2022	Iran	1	32	F (1)	Astrazeneca (1)	Myocarditis (1)	Discharged (1)
Hinton et al., [41]	2021	UK	1	38	M (1)	Pfizer (1)	Coronary thrombosis (1)	Discharged (1)
Hryniewicki et al., [42]	2022	USA	1	18	M (1)	Pfizer (1)	Pericarditis	Discharged (1)
Huang et al., [43]	2022	Taiwan	3	78-89	M (1); F (2)	Astrazeneca (3)	Myocardial infarction (3)	Discharged (3)
Hung et al., [44]	2021	Taiwan	1	23	M (1)	Astrazeneca (1)	Myopericarditis (1)	Discharged (1)
Imran et al., [45]	2022	USA	1	30	M (1)	Unspecific mRNA vaccine (1)	Myocardial fibrosis (1)	Discharged (1)
Iqbal et al., [46]	2022	Pakistan	1	61	M (1)	Moderna (1)	Myocardial infarction (1)	Discharged (1)
Issak et al., [47]	2021	Germany	1	15	M (1)	Pfizer (1)	Myocarditis (1)	Discharged (1)
Iwamuro et al., [48]	2022	Japan	1	17	M (1)	Moderna (1)	Myocarditis (1)	Discharged (1)
Jani et al., [49]	2021	UK	1	65	F (1)	Moderna (1)	Takotsubo Cardiomyopathy (1)	Discharged (1)
Javed et al., [50]	2022	USA	1	64	M (1)	Moderna (1)	Hemorrhagic pleuropericarditis (1)	Discharged (1)
Kadwalwala et al., [51]	2021	USA	1	38	M (1)	Moderna (1)	Myocarditis and cardiogenic shock (1)	Discharged (1)
Kaneta et al., [52]	2022	Japan	1	25	M (1)	Moderna (1)	Myocarditis (1)	Discharged (1)
Kang et al., [53]	2022	South Korea	1	48	F (1)	Pfizer (1)	Myocarditis (1)	Discharged (1)
Kaul et al., [54]	2021	USA	2	21-28	M (2)	Pfizer (1); Moderna (1)	Myocarditis (2)	Discharged (2)
Kawakami et al., [55]	2022	Japan	1	45	F (1)	Moderna (1)	Myocarditis (1)	Discharged (1)
Kawano et al., [56]	2022	Japan	1	60	F (1)	Pfizer (1)	Myocarditis (1)	Discharged (1)
Kazama et al., [57]	2022	Japan	1	48	F (1)	Moderna (1)	Myocarditis (1)	Discharged (1)
Khogali et al., [58]	2021	Qatar	1	29	F (1)	Moderna (1)	Perimyocarditis (1)	Discharged (1)
Kim HW et al., [59]	2021	South Korea	4	23-70	M (3); F (1)	Pfizer (2); Moderna (2)	Myocarditis (4)	Discharged (4)
Kim IC et al., [60]	2021	South Korea	1	24	M (1)	Pfizer (1)	Myocarditis (1)	Not described (1)
Kim DW et al., [61]	2021	South Korea	1	29	M (1)	Pfizer (1)	Myopericarditis (1)	Discharged (1)
Kimball et al., [62]	2022	USA	1	57	M (1)	Pfizer (1)	Intermittent complete heart block with ventricular standstill (1)	Discharged (1)
Kimura et al., [63]	2022	Japan	1	69	M (1)	Pfizer (1)	Myocarditis (1)	Discharged (1)
King et al., [64]	2021	USA	4	20-30	M (3); F (1)	Pfizer (1); Moderna (3)	Myocarditis (4)	Discharged (4)
Koizumi et al., [65]	2021	Japan	2	22-27	M (2)	Moderna (2)	Myocarditis (2)	Discharged (1); Not described (1)
Kojima et al., [66]	2022	Japan	1	17	M (1)	Pfizer (1)	Myocarditis (1)	Discharged (1)
Korzeniowska et al., [67]	2022	Poland	1	65	M (1)	Pfizer (1)	Pericarditis (1)	Discharged (1)
Kumar et al., [68]	2021	India	2	60-75	M (2)	Astrazeneca (2)	Myocardial infarction (2)	Recovering (2)
Larson et al., [69]	2021	USA	8	21-56	M (8)	Pfizer (5); Moderna (3)	Myocarditis (8)	Discharged (8)
Lazaros et al., [70]	2021	Greece	8	46-76	M (3); F (5)	Pfizer (5); Moderna (1);	Pericarditis (8)	Recovering (8)

						Astrazeneca (2)		
Lee et al., [71]	2021	Singapore	3	44-73	F (3)	Pfizer (3)	Myocardial injury (1); Cardiomyopathy (1); Myocardial infarction (1)	Not described (3)
Levin et al., [72]	2021	Israel	7	18-24	M (7)	Pfizer (7)	Myocarditis (7)	Discharged (7)
Li et al., [73]	2021	China	1	31	M (1)	Sinopharm (1)	Atrial fibrillation (1)	Not described (1)
Lim et al., [74]	2021	South Korea	1	36	F (1)	Pfizer (1)	Myocarditis (1)	Discharged (1)
Loch et al., [75]	2021	Malaysia	1	34	M (1)	Pfizer (1)	Myocarditis with intraventricular thrombus (1)	Discharged (1)
Mansour et al., [76]	2021	USA	2	21-25	M (1); F (1)	Moderna (2)	Myocarditis (2)	Discharged (2)
Marshall et al., [77]	2021	USA	7	14-19	M (7)	Pfizer (7)	Myocarditis (4); Myopericarditis (3)	Discharged (7)
Marsukjai et al., [78]	2022	Thailand	1	55	F (1)	Astrazeneca (1)	Myocarditis (1)	Discharged (1)
Matta et al., [79]	2021	USA	1	27	M (1)	Pfizer (1)	Myocarditis (1)	Discharged (1)
McLean et al., [80]	2021	USA	1	16	M (1)	Pfizer (1)	Myopericarditis (1)	Discharged (1)
Mengesha et al., [81]	2022	Israel	1	43	F (1)	Pfizer (1)	Myocarditis (1)	Discharged (1)
Mimouni et al., [82]	2022	Morocco	1	14	Not described (1)	Unspecific mRNA vaccine (1)	Myocarditis (1)	Discharged (1)
Minocha et al., [83]	2021	USA	1	17	M (1)	Pfizer (1)	Recurrent Myocarditis (1)	Discharged (1)
Miqdad et al., [84]	2021	Saudi Arabia	1	18	M (1)	Pfizer (1)	Myocarditis (1)	Discharged (1)
Mishra et al., [85]	2021	India	1	68	M (1)	Astrazeneca (1)	Myocardial Infarction (1)	Discharged (1)
Misumi et al., [86]	2021	Japan	1	59	M (1)	Pfizer (1)	Pericarditis (1)	Discharged (1)
Mizoguchi et al., [87]	2022	Japan	1	88	F (1)	Pfizer (1)	Pleuropericarditis (1)	Discharged (1)
Mouch et al., [88]	2021	Israel	6	16-45	M (6)	Pfizer (6)	Myocarditis (6)	Discharged (6)
Murakami et al., [89]	2022	Japan	2	27-38	M (2)	Pfizer (2)	Myocarditis (2)	Discharged (2)
Muthukumar et al., [90]	2021	USA	1	52	M (1)	Moderna (1)	Myocarditis (1)	Discharged (1)
Nagasaka et al., [91]	2022	Japan	1	23	M (1)	Pfizer (1)	Myocarditis (1)	Discharged (1)
Naghashzadeh et al., [92]	2022	Iran	1	29	M (1)	Sputnik V (1)	Myocarditis (1)	Discharged (1)
Nakanishi et al., [93]	2022	Japan	1	70	F (1)	Unspecific mRNA vaccine (1)	Pericarditis (1)	Discharged (1)
Nassar et al., [94]	2021	USA	1	70	F (1)	Janssen (1)	Myocarditis (1)	Died (1)
Ohnishi et al., [95]	2022	Japan	1	26	M (1)	Pfizer (1)	Myocarditis (1)	Discharged (1)
Oka et al., [96]	2022	Japan	1	50	M (1)	Pfizer (1)	Myocarditis (1)	Discharged (1)
Olagunju et al., [97]	2022	USA	1	19	M (1)	Moderna (1)	Myocarditis (1)	Discharged (1)
Olmos et al., [98]	2022	Canada	1	49	F (1)	Pfizer (1)	Myocarditis (1)	Transferred to rehabilitation centre (1)
Ozdemir et al., [99]	2021	Turkey	1	41	F (1)	Sinovac (1)	Type 1 Kounis syndrome (1)	Discharged (1)
Panthong et al., [100]	2022	Thailand	2	48-50	M (2)	Sinovac (2)	Myocardial Infarction (2)	Discharged (2)
Parmar et al.,[101]	2022	USA	4	19-53	M (3); F (1)	Moderna (4)	Myocarditis (4)	Discharged (4)
Patel et al., [102]	2021	USA	5	19-37	M (5)	Pfizer (4); Moderna (1)	Myocarditis/myopericarditis (5)	Discharged (5)
Perna et al., [103]	2022	Italy	1	30	M (1)	Unspecific mRNA vaccine (1)	Pericarditis (1)	Discharged (1)

Puchalski et al., [104]	2022	Poland	5	15-17	M (5)	Pfizer (5)	Myocarditis (5)	Discharged (5)
Rosner et al., [105]	2021	USA	6	19-39	M (6)	Pfizer (4); Moderna (1); Janssen (1)	Myocarditis (6)	Discharged (1)
Sanci et al., [106]	2022	Turkey	1	22	F (1)	Pfizer (1)	Type 1 Kounis syndrome (1)	Discharged (1)
Sano et al., [107]	2022	Japan	1	20	M (1)	Moderna (1)	Myocarditis (1)	Discharged (1)
Schauer et al., [108]	2021	USA	13	age 12-17	M (12); F (1)	Pfizer (13)	Myopericarditis (13)	Discharged (13)
Schmitt et al., [109]	2021	France	1	19	M (1)	Pfizer (1)	Myocarditis (1)	Discharged (1)
Sciaccaluga et al., [110]	2022	Italy	2	20-21	M (2)	Moderna (2)	Myopericarditis (2)	Discharged (2)
Sharbatdaran et al., [111]	2022	Gredana	1	25	M (1)	Moderna (1)	Myopericarditis (1)	Discharged (1)
Shiyovich et al., [112]	2022	Israel	4	18-44	M (3); F (1)	Pfizer (4)	Myocarditis (4)	Not described (4)
Shumkova et al., [113]	2021	Bulgaria	1	23	M (1)	Pfizer (1)	Myocarditis (1)	Discharged (1)
Singh et al., [114]	2021	USA	1	24	M (1)	Pfizer (1)	Myocarditis (1)	Discharged (1)
Snapiri et al., [115]	2021	Israel	7	16-17	M (7)	Pfizer (7)	Transient cardiac injury (7)	Dishcarged (1)
Sokolska et al., [116]	2021	Poland	1	21	M (1)	Pfizer (1)	Myocarditis (1)	Not described (1)
Sonaglioni et al., [117]	2021	Italy	1	54	F (1)	Pfizer (1)	Pericarditis (1)	Not described (1)
Starekova et al., [118]	2021	USA	5	17-38	M (4); F (1)	Pfizer (3); Moderna (2)	Myocarditis (5)	Not described (5)
Stewart et al., [119]	2022	UK	1	50	F (1)	Astrazeneca (1)	Takotsubo Cardiomyopathy (1)	Discharged (1)
Sulemankhil et al., [120]	2022	USA	1	33	M (1)	Janssen (1)	Myocarditis (1)	Discharged (1)
Sung et al., [121]	2021	USA	2	42-68	M (1); F (1)	Moderna (2)	Myocardial Infarction (2)	Discharged (2)
Tailor et al., [122]	2021	USA	1	44	M (1)	Moderna (1)	Myocarditis (1)	Discharged (1)
Tano et al., [123]	2021	USA	6	15-17	M (6)	Pfizer (6)	Perimyocarditis (6)	Discharged (5); Not described (1)
Tedeschi et al., [124]	2021	Italy	1	17	F (1)	Pfizer (1)	Takotsubo syndrome (1)	Discharged (1)
Tinoco et al., [125]	2021	Portugal	1	39	M (1)	Pfizer (1)	Perimyocarditis (1)	Discharged (1)
Toida et al., [126]	2022	Japan	1	80	F (1)	Pfizer (1)	Takotsubo syndrome (1)	Discharged (1)
Ujueta et al., [127]	2021	USA	1	62	F (1)	Janssen (1)	Myocarditis (1)	Died (1)
Verma et al., [128]	2021	USA	2	42-45	M (1); F (1)	Pfizer (2)	Myocarditis (2)	Discharged (1); Died (1)
Vidula et al., [129]	2021	USA	5	18-60	M (2); F (3)	Pfizer (4); Moderna (1)	Myocarditis (2); Pericarditis (2); Stress cardiomyopathy (1)	Discharged (3); Not described (2)
Watanabe et al., [130]	2022	Japan	4	19-48	M (4)	Pfizer (1); Moderna (3)	Myocarditis (4)	Discharged (4)
Williams et al., [131]	2021	Canada	1	34	M (1)	Moderna (1)	Myocarditis (1)	Discharged (1)
Yamaura et al., [132]	2022	Japan	1	30	F (1)	Pfizer (1)	Takotsubo cardiomyopathy (1)	Discharged (1)
Yen et al., [133]	2022	Taiwan	1	32	M (1)	Moderna (1)	Myocarditis (1)	Discharged (1)
Zaki et al., [134]	2022	Qatar	1	55	M (1)	Pfizer (1)	Pericarditis and pericardial effusion (1)	Discharged (1)

Supplementary S4: Detailed description of studies reporting cases with neurological complications following COVID-19 vaccination (n = 245).

**Author**	**Year**	**Country**	**Number of cases**	**Age range**	**Gender**	**Vaccines**	**Diagnosis**	**Outcome**
Abicic et al., [1]	2021	Croatia	1	24	F (1)	Pfizer (1)	Miller Fisher Syndrome (1)	Discharged (1)
Ahmad et al., [2]	2021	Iraq	1	62	F (1)	Pfizer (1)	Aseptic Meningitis (1)	Discharged (1)
Alabkal et al., [3]	2021	Canada	1	26	F (1)	Pfizer (1)	Incomplete Subacute Transverse Myelitis (1)	Discharged (1)
Aladdin et al.,[4]	2021	Saudi Arabia	1	42	F (1)	Astrazeneca (1)	Refractory Status Epilepticus (1)	Discharged (1)
Albokhari et al., [5]	2021	Saudi Arabia	1	16	F (1)	Pfizer (1)	Acute Inflammatory Transverse Myelitis (1)	Discharged (1)
Alhashim et al., [6]	2022	Saudi Arabia	1	28	F (1)	Pfizer (1)	Cerebral Venous Sinus Thrombosis (1)	Discharged (1)
Allen et al., [7]	2021	UK	4	20-57	M (4)	Astrazeneca (4)	Guillain Barre Syndrome (4)	Not described (4)
Al-Mashdali et al., [8]	2021	Qatar	1	32	M (1)	Moderna (1)	Acute Hyperactive Encephalopathy (1)	Discharged (1)
Al-Mayhani et al.,[9]	2021	UK	3	35-43	M (1); F (2)	Astrazeneca (3)	Ischaemic Stroke (3)	Brainstem death (1); Recovering (1); Critically ill (1)
Al-Midfai et al., [10]	2022	USA	1	25	F (1)	Janssen (1)	Acute Multiple Sclerosis Exacebation (1)	Discharged (1)
Al-Quliti et al., [11]	2022	Saudi Arabia	1	56	F (1)	Astrazeneca (1)	Acute Demyelinating Encephalomyelitis (1)	Discharged (1)
Alsallamin et al., [12]	2022	USA	1	59	M (1)	Not described (1)	Cerebral Vein Thrombosis (1)	Discharged (1)
Alshararni et al., [13]	2021	Saudi Arabia	1	38	M (1)	Pfizer (1)	Acute Transverse Myelitis (1)	Not described (1)
Aly et al., [14]	2022	Egypt	1	29	M (1)	Astrazeneca (1)	Guillain Barre Syndrome (1)	Discharged (1)
Amjad et al., [15]	2022	USA	1	78	M (1)	Pfizer (1)	Parsonage Turner Syndrome (1)	Discharged (1)
Anamnart et al., [16]	2022	Thailand	2	26-46	F (2)	Astrazeneca (1); Sinovac (1)	Neuromyelitis Optica Spectrum Disorder (2)	Not described (2)
Ancau et al., [17]	2022	Germany	3	25-61	M (1); F (2)	Astrazeneca (3)	Acute Hemorrhagic Encephalomyelitis (3)	Discharged (2); Died (1)
Andreozzi et al., [18]	2022	Italy	2	43-59	M (1); F (1)	Astrazeneca (2)	Facial Palsy (2)	Discharged (1); Not described (1)
Aravinda et al., [19]	2021	India	1	73	M (1)	Astrazeneca (1)	Guillain Barre Syndrome (1)	Discharged (1)
Asif et al., [20]	2022	USA	1	28	M (1)	Janssen (1)	Cerebral Venous Sinus Thrombosis (1)	Discharged (1)
Assiri et al., [21]	2022	Saudi Arabia	18	23-80	M (9); F (9)	Pfizer (8); Astrazeneca (10)	Optic neuritis (2); Cerebral Venous thrombosis (1); Cerebral Venous thrombosis and intracranial hemorrhage (2); Guillain Barre Syndrome (1); Ischemic Cerebrovascular accident (7); Ischemic Cerebrovascular accident	Discharged (12); Died (2); Admitted to extended care (4)

							and Vestibular neuritis (1); Miller Fisher Syndrome (1); Seizure (1); Seizure and encephalopathy (1); Ischemic Attack (1)	
Azam et al.,[22]	2021	UK	1	67	M (1)	Astrazeneca (1)	Guillain Barre Syndrome (1)	Not described (1)
Bagella et al., [23]	2021	Italy	1	49	M (1)	Astrazeneca (1)	Chronic Inflammatory Demyelinating Polyneuropathy (1)	Not described (1)
Ballout et al., [24]	2022	USA	4	27-81	M (1); F (3)	Pfizer (2); Moderna (2)	Neuromyelitis Optica Spectrum Disorder (2); Acute Disseminated Encephalomyelitis (1); Meningoencephalitis (1)	Discharged (2); Died (1); Not described (1)
Balloy et al., [25]	2022	France	1	63	M (1)	Astrazeneca (1)	Neuralgic amyotrophy (1)	Discharged (1)
Bax et al., [26]	2021	Italy	2	51-90	M (1); F (1)	Unspecific Adenoviral vector vaccine (1); Unspecific mRNA vaccine (1)	Acute motor sensory axonal neuropathy (1); Acute Inflammatory demyelinating polyneuropathy (1)	Not described (2)
Bazrafshan et al., [27]	2022	Iran	1	68	F (1)	Astrazeneca (1)	Guillain Barre Syndrome (1)	Discharged (1)
Bensaidane et al., [28]	2022	Canada	1	56	M (1)	Astrazeneca (1)	Acute Necrotizing encephalopathy (1)	Discharged (1)
Berlot et al., [29]	2022	Italy	1	69	F (1)	Astrazeneca (1)	Cerebral Venous Sinus Thrombosis (1)	Recovering (1)
Bernheimer et al., [30]	2022	USA	1	42	F (1)	Moderna (1)	Parsonage Turner Syndrome (1)	Discharged (1)
Biswas et al., [31]	2021	India	1	49	M (1)	Astrazeneca (1)	Guillain Barre Syndrome (1)	Discharged (1)
Bogs et al., [32]	2022	Germany	1	15	M (1)	Pfizer (1)	Aseptic Meningitis (1)	Discharged (1)
Bollo et al., [33]	2021	Italy	1	42	F (1)	Moderna (1)	Guillain Barre Syndrome (1)	Discharged (1)
Bonifacio et al., [34]	2022	UK	4	43-66	M (4)	Astrazeneca (4)	Guillain Barre Syndrome (4)	Recovering (4)
Borelli et al., [35]	2021	Brazil	1	42	M (1)	Astrazeneca (1)	Optic Neuritis and Longitudinally Extensive Transverse Myelitis (1)	Discharged (1)
Bouattour et al., [36]	2022	Tunisia	1	67	M (1)	Pfizer (1)	Guillain Barre Syndrome (1)	Discharged (1)
Bramer et al., [37]	2022	UK	1	50	F (1)	Pfizer (1)	Vestibular Neuronitis (1)	Discharged (1)
Burillo et al., [38]	2021	Spain	1	38	M (1)	Astrazeneca (1)	Amyotrophic Neuralgia (1)	Not described (1)
Burrows et al., [39]	2021	UK	1	61	M (1)	Pfizer (1)	Bells Palsy (1)	Discharged (1)
Butler et al., [40]	2021	UK	2	36-38	F (2)	Pfizer (1); Moderna (1)	Functional Neurological Disorders (2)	Discharged (1); Not described (1)
Cabral et al., [41]	2022	Portugal	1	33	M (1)	Pfizer (1)	Transverse Myelitis (1)	Discharged (1)
Cao et al., [42]	2022	China	1	24	F (1)	Sinopharm (1)	Acute Disseminated Encephalomyelitis (1)	Not described (1)
Carneiro et al., [43]	2022	Portugal	1	62	F (1)	Pfizer (1)	Aseptic Meningitis (1)	Discharged (1)
Carranza et al., [44]	2022	USA	1	53	F (1)	Janssen (1)	Guillain Barre Syndrome (1)	Discharged (1)
Castan et al., [45]	2022	France	1	57	M (1)	Janssen (1)	Ischemic Stroke (1)	Discharged (1)
Castiglione et al., [46]	2022	Argentina	9	39-87	M (7); F (2)	Astrazeneca (4); Sputnik V (5)	Guillain Barre Syndrome (9)	Recovering (8); Died (1)
Cellina et al., [47]	2022	Italy	1	35	F (1)	Moderna (1)	Bells Palsy (1)	Recovering (1)
Censcak et al., [48]	2021	Czech	1	42	M (1)	Pfizer (1)	Guillain Barre Syndrome (1)	Not described (1)
Chakrabarti et al.,	2022	India	1	60	F (1)	Astrazeneca (1)	Progressive dementia with asymmetric ridigity (1)	Died (1)

[49]								
Chan et al., [50]	2022	Singapore	2	38-43	F (2)	Pfizer (2)	Aseptic Meningitis (2)	Discharged (2)
Chaurasia et al., [51]	2022	Nepal	1	65	M (1)	Astrazeneca (1)	Cognitive Deficit and Memory Impairments (1)	Discharged (1)
Chen et al., [52]	2021	China	1	Not described	F (1)	Unspecific inactivated virus vaccine (1)	Neuromyelitis Optica Spectrum Disorder (1)	Not described (1)
Chua et al., [53]	2022	USA	1	64	M (1)	Moderna (1)	Parsonage Turner Syndrome (1)	Recovering (1)
Chun et al., [54]	2022	South Korea	2	76-80	F (2)	Pfizer (2)	Guillain Barre Syndrome (1); Acute Inflammatory Demyelinating Polyneuropathy (1)	Transferred to Rehabilitation centre (2)
Cicalese et al.,[55]	2022	Italy	1	88	M (1)	Moderna (1)	Third Cranial Nerve Palsy (1)	Discharged (1)
Colella et al., [56]	2021	Italy	1	37	M (1)	Pfizer (1)	Bells Palsy (1)	Discharged (1)
Consoli et al., [57]	2022	Italy	1	37	F (1)	Pfizer (1)	Status Migrainosus (1)	Not described (1)
Correa et al., [58]	2021	Brazil	3	42-65	M (3)	Astrazeneca (3)	Ischemic Stroke (1); Facial Nerve Palsy (1); Cervical Myelitis (1)	Discharged (2); Not described (1)
Costentin et al., [59]	2021	France	1	26	F (1)	Astrazeneca (1)	Acute Ischemic Stroke (1)	Not described (1)
Daher et al., [60]	2022	Lebanon	1	46	F (1)	Unspecific mRNA vaccine (1)	Unilateral Lower Limb Paralysis (1)	Discharged (1)
Dalwadi et al., [61]	2021	USA	1	86	F (1)	Moderna (1)	Axonal variant Gullain-Barre syndrome (1)	Not described (1)
Dang et al.,[62]	2021	Australia	1	63	M (1)	Astrazeneca (1)	Miller Fisher Syndrome and Guillain Barre Syndrome (1)	Discharged (1)
Desai et al., [63]	2021	USA	1	69	F (1)	Moderna (1)	Myasthenia Gravis Exacerbation	Recovering (1)
Diaz-Segarra et al., [64]	2021	USA	1	35	F (1)	Pfizer (1)	Parsonage Turner Syndrome (1)	Not described (1)
Doi et al., [65]	2022	Japan	1	27	F (1)	Pfizer (1)	Cervical Transverse Myelitis (1)	Discharged (1)
Einstein et al., [66]	2021	USA	1	58	F (1)	Not described (1)	Bells Palsy (1)	Not described (1)
Eom et al., [67]	2022	South Korea	2	23-81	M (1); F (1)	Pfizer (2)	Acute Transverse Myelitis (2)	Discharged (2)
Erdem et al.,[68]	2021	Turkey	1	78	F (1)	Sinovac (1)	Acute Transverse Myelitis (1)	Discharged (1)
Etemadifar et al.,[69]	2021	Iran	1	34	F (1)	Sputnik V (1)	Relapse of Multiple Sclerosis (1)	Discharged (1)
Fan et al., [70]	2022	Taiwan	1	22	M (1)	Moderna (1)	Encephalitis and Statis Epilepticus (1)	Recovering (1)
Fernandes et al., [71]	2022	Trinidad and Tobago	4	16-61	M (2); F (2)	Pfizer (2); Astrazeneca (2)	New Onset Seizures (1); Longitudinally Extensive Transverse Myelitis (1); Guillain Barre Syndrome (1); Meningitis Retention Syndrome (1)	Discharged (1)
Finsterer et al., [72]	2021	Austria	1	32	M (1)	Unspecific Vector-based vaccine	Guillain Barre Syndrome (1)	Recovering (1)
Franco et al., [73]	2022	Spain	2	53-65	M (2)	Pfizer (2)	Ischemic Optic Neuropathy (2)	Discharged (2)
Fujimori et al., [74]	2021	Japan	1	40	F (1)	Pfizer (1)	Multiple Sclerosis (1)	Not described (1)
Fukushima et al., [75]	2022	Japan	1	55	F (1)	Pfizer (1)	Sensory Ataxic subtype of Guillain Barre Syndrome (1)	Transferred to Rehabilitation centre (1)
Galvez et al., [76]	2021	Colombia	1	73	M (1)	Sinovac (1)	Guillain Barre Syndrome (1)	Discharged (1)
Gama et al., [77]	2022	Brazil	1	47	F (1)	Astrazeneca (1)	Longitudinal Extensive Transverse Myelitis (1)	Discharged (1)

Gao et al., (a)[78]	2021	Taiwan	1	76	F (1)	Moderna (1)	Acute Transverse Myelitis (1)	Discharged (1)
Gao et al., (b)[79]	2022	Taiwan	1	82	F (1)	Moderna (1)	Acute Encephalitis (1)	Discharged (1)
Garcia-Grimshaw et al., [80]	2021	Mexico	6	25-81	M (4); F (2)	Pfizer (6)	Guillain Barre Syndrome (6)	Discharged (5); Died (1)
Garg et al., [81]	2022	India	1	56	F (1)	Astrazeneca (1)	Tumefactive Demyelinating Brain Lesion (1)	Discharged (1)
George et al., [82]	2022	USA	1	60	M (1)	Moderna (1)	Guillain Barre Syndrome (1)	Discharged (1)
Ghosh et al., [83]	2021	India	1	68	M (1)	Astrazeneca (1)	Focal non-motor onset seizure with impaired awareness (1)	Discharged (1)
Grenacher et al., [84]	2021	Germany and Austria	9	Unknown (9)	Unknown (9)	Astrazeneca (9)	Cerebral Venous Thrombosis (9)	Died (5); Recovering (3); Not described (1)
Guarnaccia et al., [85]	2022	USA	1	32	F (1)	Pfizer (1)	Incomplete transverse myelitis secondary to multiple sclerosis (1)	Recovering (1)
Guditi et al., [86]	2021	India	1	18	F (1)	Astrazeneca (1)	Cerebral Thrombotic Thrombocytopenia (1)	Died (1)
Hasan et al., [87]	2021	UK	1	62	F (1)	Astrazeneca (1)	Guillain Barre Syndrome (1)	Critically ill (1)
Havla et al., [88]	2022	Germany	1	28	F (1)	Pfizer (1)	Multiple Sclerosis (1)	Recovering (1)
Hemalatha et al., [89]	2021	India	1	43	F (1)	Astrazeneca (1)	Guillain Barre Syndrome (1)	Transferred to Rehabilitation centre (1)
Hirose et al., [90]	2021	Japan	1	70	M (1)	Moderna (1)	Acute Autoimmune Transverse Myelitis (1)	Discharged (1)
Hsiao et al., [91]	2021	Taiwan	1	41	M (1)	Astrazeneca (1)	Acute Transverse Myelitis (1)	Discharged (1)
Hughes et al., [92]	2021	USA	1	65	M (1)	Pfizer (1)	Guillain Barre Syndrome (1)	Discharged (1)
Iftikhar et al., [93]	2021	Qatar	1	36	M (1)	Moderna (1)	Bells Palsy (1)	Discharged (1)
Introna et al., [94]	2021	Italy	1	62	M (1)	Astrazeneca (1)	Guillain Barre Syndrome (1)	Recovering (1)
Ish et al., [95]	2021	India	1	50	M (1)	Covaxin (1)	Facial Nerve Palsy (1)	Discharged (1)
James et al., [96]	2021	India	3	54-66	M (2); F (1)	Astrazeneca (3)	Guillain Barre Syndrome (3)	Discharged (1); Not described (2)
John et al., [97]	2021	India	1	55	F (1)	Astrazeneca (1)	Guillain Barre Syndrome (1)	Discharged (1)
Kanabar et al., [98]	2021	UK	2	56-61	M (1); F (1)	Astrazeneca (2)	Guillain Barre Syndrome (2)	Recovering (2)
Kang et al., [99]	2022	South Korea	1	32	M (1)	Pfizer (1)	Aseptic Meningitis (1)	Discharged (1)
Kania et al., [100]	2021	Poland	1	19	F (1)	Moderna (1)	Acute Disseminated Encephalomyelitis (1)	Discharged (1)
Karimi et al., [101]	2021	Iran	8	26-87	M (6); F (2)	Astrazeneca (1); Sinopharm (3); Sputnik V (4)	Guillain Barre Syndrome (6); Acute Motor Axonal Neuropathy subtype of Guillain Barre Syndrome (2)	Discharged (7); Recovering (1)
Katada et al., [102]	2022	Japan	1	44	F (1)	Pfizer (1)	Chronic Inflammatory Demyelinating Polyneuropathy (1)	Not described (1)
Kataria et al., [103]	2022	USA	1	57	F (1)	Pfizer (1)	Relapse of Multiple Sclerosis (1)	Discharged (1)
Kavya et al., [104]	2021	India	1	19	M (1)	Astrazeneca (1)	Acute Disseminated Encephalomyelitis (1)	Discharged (1)
Kawtharani et al., [105]	2021	Lebanon	1	37	F (1)	Astrazeneca (1)	Longitudinal Extensive Transverse Myelitis (1)	Discharged (1)
Kaya et al., [106]	2022	Turkey	1	45	F (1)	Pfizer (1)	Trigeminal Neuralgia (1)	Discharged (1)
Keir et al.,[107]	2021	USA	1	57	F (1)	Pfizer (1)	Neurologic Phantosmia (1)	Not described (1)
Kenda et al., [108]	2021	Slovenia	1	51	F (1)	Astrazeneca (1)	Acute Ischemic Stroke (1)	Discharged (1)

Khadka et al., [109]	2021	Nepal	1	44	M (1)	Janssen (1)	Acute Inflammatory Demyelinating Polyneuropathy (1)	Discharged (1)
Khajavirad et al., [110]	2022	Iran	2	56-70	F (2)	Astrazeneca (2)	Encephalopathy (1); Cerebral Venous Sinus Thrombosis (1)	Discharged (2)
Khan et al., [111]	2022	India	1	67	F (1)	Moderna (1)	Acute Tranverse Myelitis (1)	Discharged (1)
Khayat-Khoei et al., [112]	2022	USA	7	24-64	M (2); F (5)	Pfizer (4); Moderna (3)	Multiple sclerosis exacerbation (3); Relapsing-remitting Multiple sclerosis (3); Neuromyelitis Optica (1)	Discharged (4); Not described (3)
Kilic et al., [113]	2021	Turkey	1	34	M (1)	Pfizer (1)	New Onset Refractory Status Epilepticus (1)	Discharged (1)
Kim JE et al., (b)[114]	2022	South Korea	13	18-84	M (4); F (9)	Pfizer (5); Astrazeneca (8)	Guillain Barre Syndrome (5); Paraparetic Guillain Barre Syndrome (2); Acute Motor Axonal Neuropathy subtype of Guillain Barre Syndrome (3); Acute Inflammatory Demyelinating Polyradiculoneuropathy subtype of Guillain-Barre syndrome (3)	Discharged (13)
Kim JW et al., (c.)[115]	2022	South Korea	2	42-48	M (1); F (1)	Pfizer (1); Astrazeneca (1)	Guillain Barre Syndrome (2)	Discharged (2)
Kim NK et al., (d)[116]	2022	South Korea	1	21	M (1)	Pfizer (1)	Guillain Barre Syndrome (1)	Discharged (1)
Kim SI et al., (a)[117]	2021	South Korea	1	45	F (1)	Astrazeneca (1)	Leg paralysis Neuralgic Amyotrophy (1)	Discharged (1)
Kim YS et al., (e.)[118]	2022	USA	1	16	F (1)	Pfizer (1)	Sensory Predominant Guillain Barre Syndome (1)	Discharged (1)
Kobayashi et al., [119]	2022	Japan	1	46	F (1)	Pfizer (1)	Encephalitis (1)	Discharged (1)
Koh et al., [120]	2021	Singapore	3	44-58	M (3)	Pfizer (2); Moderna (1)	Neuralgic Amyotrophy (3)	Discharged (3)
Kotal et al., [121]	2021	India	1	32	F (1)	Astrazeneca (1)	Cerebral Sinus Venous Thrombosis (1)	Discharged (1)
Kripalani et al.,[122]	2021	India	1	52	F (1)	Astrazeneca (1)	Guillain Barre Syndrome (1)	Transferred to Rehabilitation Centre (1)
Kubota et al., [123]	2022	Japan	1	65	M (1)	Pfizer (1)	Oculomotor Nerve Palsy (1)	Discharged (1)
Kwon et al., [124]	2022	South Korea	1	57	F (1)	Astrazeneca (1)	Autoimmune Encephalitis (1)	Recovering (1)
Kyle et al., [125]	2022	USA	1	80	F (1)	Pfizer (1)	Encephalomyelitis (1)	Died (1)
Lagosz et al., [126]	2022	Poland	1	64	M (1)	Not described (1)	Relapse of Multiple Sclerosis (1)	Recovering (1)
Lee et al., [127]	2022	South Korea	1	61	M (1)	Astrazeneca (1)	Guillain Barre Syndrome (1)	Recovering (1)
Leemans et al., [128]	2022	Belgium	8	57-81	M (6); F (2)	Pfizer (6); Astrazeneca (2)	Guillain Barre Syndrome (3); Right lower trunk Branchial Plexopathy (1); Axonal sensorimotor polyneuropathy (1); Acute Inflammatory Demyelinating Polyneuropathy (1); Chronic Inflammatory Demyelinating Polyradiculoneuropathy (2)	Discharged (7); Recovering (1)
Ling et al., [129]	2021	Canada	1	63	M (1)	Astrazeneca (1)	Relapse of Guillain Barre Syndrome (1)	Recovering (1)
Liu et al., [130]	2021	USA	2	73-86	M (1); F (1)	Moderna (2)	Encephalopathy associated with Non-convulsive focal epilepticus (2)	Discharged (2)
Lohmann et al., [131]	2022	Germany	1	68	F (1)	Pfizer (1)	AQP4-antibody positive neuromyelitis optica spectrum disorder (1)	Discharged (1)

Luca et al., [132]	2022	Italy	1	82	F (1)	Pfizer (1)	Chronic Inflammatory Axonal Polyneuropathy (1)	Transferred to Rehabilitation Centre (1)
Luciano et al., [133]	2022	Brazil	1	66	F (1)	Astrazeneca (1)	Cerebral Thrombosis (1)	Died (1)
Luitel et al., [134]	2022	Nepal	1	48	M (1)	Astraneneca (1)	Guillain Barre Syndrome (1)	Discharged (1)
Mahajan et al., [135]	2021	USA	1	50	M (1)	Pfizer (1)	Parsonage Turner Syndrome (1)	Recovering (1)
Malhotra et al., [136]	2021	India	1	36	M (1)	Astrazeneca (1)	Myelitis (1)	Discharged (1)
Malik et al., [137]	2021	USA	1	43	F (1)	Janssen (1)	Transient Ischaemic Attack (1)	Discharged (1)
Mancuso et al., [138]	2022	Italy	1	42	F (1)	Astrazeneca (1)	Arterial Intracranial Thrombosis (1)	Transferred to Rehabilitation Centre (1)
Manea et al., [139]	2022	Romania	1	29	M (1)	Pfizer (1)	Multiple Cranial Nerve Palsies (1)	Discharged (1)
Maniscalco et al., [140]	2021	Italy	1	26	F (1)	Pfizer (1)	Acute Relapse Multiple Sclerosis (1)	Discharged (1)
Maramattom et al., (a) [141]	2022	India	5	42-65	M (4); F (1)	Astrazeneca (5)	Acute Disseminated Encephalomyelitis (2); Tumefactive Demyelination (1); Limbic Encephalitis (1); Opsoclonus myoclonus ataxia syndrome (1)	Discharged (1); Recovering (4)
Maramattom et al., (b)[142]	2021	India	1	44	F (1)	Astrazeneca (1)	Cerebral Venous Sinus Thrombosis (1)	Discharged (1)
Maramattom et al., (c.)[143]	2021	India	7	43-70	M (1); F (6)	Astrazeneca (7)	Guillain Barre Syndrome (7)	Recovering (7)
Maroufi et al., [144]	2022	Iran	1	31	F (1)	Astrazeneca (1)	Longitudinal Extensive Transverse Myelitis (1)	Discharged (1)
Martin-Villares et al., [145]	2022	Spain	1	34	F (1)	Moderna (1)	Bells Palsy (1)	Discharged (1)
Masuccio et al., [146]	2021	Italy	1	Not described	M (1)	Moderna (1)	Guillain Barre Syndrome (1)	Recovering (1)
Materneh et al., [147]	2021	Qatar	1	61	M (1)	Moderna (1)	Guillain Barre Syndrome (1)	Discharged (1)
Mazzeo et al., [148]	2021	Italy	2	45-55	M (1); F (1)	Astrazeneca (2)	Cerebral Venous Sinus Thrombosis (2)	Died (2)
McKean et al., [149]	2021	Malta	1	48	M (1)	Astrazeneca (1)	Guillain Barre Syndrome (1)	Discharged (1)
Mehta et al., [150]	2021	UK	2	25-32	M (2)	Astrazeneca (2)	Cerebral Venous Sinus Thrombosis (2)	Not described (2)
Melo-Silva et al., [151]	2021	Brazil	1	57	F (1)	Astrazeneca (1)	Large Hemorrhagic Stroke (1)	Recovering (1)
Michele et al., [152]	2022	Italy	2	55-57	F (2)	Astrazeneca (2)	Cerebral Infarction (2)	Recovering (1); Brain death (1)
Min et al., [153]	2021	South Korea	2	37-58	M (1); F (1)	Astrazeneca (2)	Guillain Barre Syndrome (2)	Not described (2)
Mirmosayyed et al., [154]	2022	Iran	2	27-58	M (1); F (1)	Sputnik V (2)	Bells Palsy (2)	Discharged (1); Recovering (1)
Miyamoto et al., [155]	2022	Japan	1	54	F (1)	Pfizer (1)	Acute Disseminated Encephalomyelitis (1)	Transferred to Rehabilitation Centre (1)
Miyaue et al., [156]	2022	Japan	1	75	M (1)	Pfizer (1)	Refractory Longitudinally Extensive Transverse Myelitis (1)	Transferred to Rehabilitation

								Centre (1)
Morehouse et al., [157]	2021	USA	1	49	F (1)	Janssen (1)	Guillain Barre Syndrome (1)	Discharged (1)
Muir et al., [158]	2021	USA	1	48	F (1)	Janssen (1)	Cerebral Venous Thrombosis (1)	Critically ill (1)
Mumoli et al., [159]	2022	Italy	1	45	M (1)	Astrazeneca (1)	Acute Disseminated Encephalomyelitis anti-MOG antibody	Discharged (1)
Mussatto et al., [160]	2022	USA	1	60	M (1)	Pfizer (1)	Bells Palsy (1)	Discharged (1)
Nagalli et al., [161]	2022	USA	1	49	F (1)	Moderna (1)	Guillain Barre Syndrome (1)	Discharged (1)
Nagaratnam et al., [162]	2022	Australia	1	36	F (1)	Astrazeneca (1)	Acute Disseminated Encephalomyelitis (1)	Discharged (1)
Nasiri et al., [163]	2022	Saudi Arabia	2	27-33	M (2)	Pfizer (2)	Guillain Barre Syndome (2)	Discharged (1); Recovering (1)
Nasuelli et al., [164]	2021	Italy	1	59	M (1)	Astraneneca (1)	Guillain Barre Syndrome (1)	Recovering (1)
Nawaz et al., [165]	2022	UK	1	47	M (1)	Not described (1)	Parsonage Turner Syndrome (1)	Discharged (1)
Netravathi et al., [166]	2022	India	29	20-65	M (12); F(17)	Astrazeneca (27); Covaxin (2)	Acute Demyelinating Encephalomyelitis (3); Acute Transverse Myelitis (3); Longitudinally extensive transverse myelitis (2); Cervical Cord Demyelination (1); Tumefactive Demyelination (1); Area Postrema Syndrome - Aquaporin 4 positive NMO (1); Bilateral Optic Neuritis (1); Brainstem syndrome (1); Central Nervous System Demyelinating (2); Cervical Cord Myelopathy (1); Cervical Myelopathy - Multiple Sclerosis (1); Longitudinal Extensive Transverse Myelitis - Aquaporin 4 positive neuromyelitis optica (1); MOG associated Acute Disseminated Encephalomyelitis (3); MOG associated Longitudinally Extensive Transverse Myelitis (2); MOG associated Optic Neuritis (4); MOG associated Opticomyelopathy (1); Optic Neuritis (1)	Recovering (29)
Nishiguchi et al., [167]	2021	Japan	1	71	M (1)	Pfizer (1)	Miller Fisher Syndrome (1)	Discharged (1)
Nishizawa et al., [168]	2021	Japan	1	62	F (1)	Janssen (1)	Bells Palsy (1)	Not described (1)
Nistri et al., [169]	2021	Italy	16	30-66	M (4); F (12)	Pfizer (10); Astrazeneca (4); Moderna (2)	Multiple Sclerosis (3); Recurrent Multiple Sclerosis (13)	Discharged (3); Recovering (8); Not described (5)
Ogbebor et al., [170]	2021	USA	1	86	F (1)	Pfizer (1)	Guillain Barre Syndrome (1)	Discharged (1)
Onoda et al., [171]	2022	Japan	1	77	F (1)	Pfizer (1)	Trigeminal Neuropathy (1)	Discharged (1)
Oo et al., [172]	2021	Australia	4	51-72	M (3); F (1)	Astrazeneca (4)	Gullain Barre Syndrome (3); Chronic Inflammatory Demyelinating Polyradiculoneuropathy (1)	Transferred to Rehabilitation centre (3); Recovering (1)
Oshida et al., [173]	2022	Japan	3	44-75	F (3)	Pfizer (3)	Aneurysmal Subarachnoid Hemorrhage (3)	Discharged (1); Transferred to Rehabilitation Centre (2)
Ozgen et al., [174]	2021	Turkey	1	46	F (1)	Sinovac (1)	Acute Disseminated Encephalomyelitis (1)	Discharged (1)
Pagenkopf et al., [175]	2021	Germany	1	45	M (1)	Astrazeneca (1)	Longitudinal Extensive Transverse Myelitis (1)	Discharged (1)
Pandey et al., [176]	2022	India	1	60	F (1)	Astrazeneca (1)	Intracerebral hemorrhage (1)	Discharged (1)

Pang et al., [177]	2022	Australia	1	51	F (1)	Astrazeneca (1)	Cerebral Venous Sinus Thrombosis (1)	Discharged (1)
Patel et al., [178]	2021	UK	1	37	M (1)	Astrazeneca (1)	Guillain Barre Syndrome (1)	Recovering (1)
Permezel et al., [179]	2021	Australia	1	63	M (1)	Astrazeneca (1)	Acute Disseminated Encephalomyelitis (1)	Died (1)
Pothiawala et al., [180]	2021	Singapore	1	46	M (1)	Moderna (1)	Bells Palsy (1)	Discharged (1)
Poudel et al., [181]	2022	Nepal	1	17	F (1)	Moderna (1)	Bells Palsy (1)	Discharged (1)
Prasad et al., [182]	2021	USA	1	41	M (1)	Janssen (1)	Bifacial Diplegia variant of Guillain Barre Syndrome (1)	Transferred to Rehabilitation Centre (1)
Queler et al., [183]	2022	USA	2	44-49	M (2)	Pfizer (1); Moderna (1)	Parsonage Turner Syndrome (2)	Discharged (2)
Quintanilla-Bordas et al., [184]	2022	Spain	3	16-41	F (3)	Pfizer (1); Moderna (2)	Multiple Sclerosis (3)	Recovering (3)
Rao et al., [185]	2021	USA	1	42	F (1)	Pfizer (1)	Guillain Barre Syndrome (1)	Discharged (1)
Rastogi et al., [186]	2022	Canada	1	59	F (1)	Moderna (1)	Encephalitis (1)	Discharged (1)
Razok et al., [187]	2021	Qatar	1	73	M (1)	Pfizer (1)	Guillain Barre Syndrome (1)	Discharged (1)
Repajic et al., [188]	2021	USA	1	57	F (1)	Pfizer (1)	Recurrent Bells Palsy (1)	Recovering (1)
Reyes-Capo et al., [189]	2021	USA	1	59	F (1)	Pfizer (1)	Acute Abducens Nerve Palsy (1)	Not described (1)
Rinaldi et al., [190]	2021	Italy	1	45	M (1)	Astrazeneca (1)	Acute Disseminated Encephalomyelitis (1)	Discharged (1)
Rizvi et al., [191]	2022	India	1	28	F (1)	Astrazeneca (1)	Acute Hemorrhagic Leukoencephalitis (1)	Discharged (1)
Rizzo et al., [192]	2022	Italy	1	58	F (1)	Janssen (1)	Ischemic Stroke (1)	Recovering (1)
Roncati et al., [193]	2022	Italy	1	28	M (1)	Moderna (1)	Cubital Tunnel Syndrome (1)	Recovering (1)
Rossetti et al., [194]	2021	USA	1	38	M (1)	Janssen (1)	Facial Diplegia Subtype of Guillain Barre Syndrome (1)	Discharged (1)
Saito et al., [195]	2021	Japan	1	42	F (1)	Pfizer (1)	Aseptic Meningitis (1)	Discharged (1)
Salbas et al., [196]	2021	Turkey	2	33-49	M (1); F (1)	Pfizer (2)	Facial Paralysis (2)	Discharged (2)
Saleh et al., [197]	2022	Germany	1	42	F (1)	Astrazeneca (1)	Vaccine Induced Immune Thrombotic Thrombocytopenia with Cerebral Venous Sinus Thrombosis (1)	Discharged (1)
Sanjay et al., [198]	2022	India	1	50	F (1)	Pfizer (1)	Non-Artheritic Anterior Ischaemic Optic Neuropathy (1)	Discharged (1)
Sansen et al., [199]	2021	Belgium	1	65	M (1)	Pfizer (1)	Miller Fisher Syndrome (1)	Recovering (1)
Saraswat et al., [200]	2021	India	1	29	M (1)	Astrazeneca (1)	Guillain Barre Syndrome (1)	Discharged (1)
Scendoni et al., [201]	2021	Italy	1	82	F (1)	Pfizer (1)	Guillain Barre Syndrome (1)	Transferred to Rehabilitation Centre (1)
Schultz et al., [202]	2021	Norway	4	37-54	F (4)	Astrazeneca (4)	Cerebellar Hemorrhagic Infarction (2); Cerebral Hemorrhage (1); Cerebral Vein Thrombosis (1)	Discharged (1); Died (3)
Senda et al.,[203]	2022	Japan	1	72	F (1)	Pfizer (1)	Acute Meningoencephalitis (1)	Discharged (1)
Sepahvand et al., [204]	2022	Iran	1	71	M (1)	Sinopharm (1)	Cervical Longitudinally Extensive Myelitis (1)	Recovering (1)
Shalilahmadi et al., [205]	2021	Iran	1	29	F (1)	Sputnik V (1)	Acute Disseminated Encephalomyelitis (1)	Recovering (1)

Sharma et al., [206]	2022	India	1	34	M (1)	Astrazeneca (1)	Brachial Plexus Neuropraxia (1)	Discharged (1)
Shields et al.,[207]	2022	USA	6	36-84	M (3); F (3)	Pfizer (4); Moderna (2)	Parsonage Turner Syndrome (6)	Discharged (6)
Shimizu et al., [208]	2021	Japan	1	88	F (1)	Pfizer (1)	Acute Disseminated Encephalomyelitis (1)	Recovering (1)
Siddiqi et al., [209]	2022	Pakistan	1	53	M (1)	Sinovac (1)	Miller Fisher Syndrome (1)	Discharged (1)
Silva et al., (a)[210]	2021	Brazil	1	62	F (1)	Astrazeneca (1)	Guillain Barre Syndrome (1)	Transferred to Rehabilitation Centre (1)
Silva et al., (b)[211]	2022	Brazil	1	27	F (1)	Astrazeneca (1)	Transverse Myelitis (1)	Discharged (1)
Singh et al., [212]	2022	USA	1	66	F (1)	Moderna (1)	Chronic Inflammatory Demyelinating Polyneuropathy (1)	Transferred to Rehabilitation Centre (1)
Siriratnam et al., [213]	2022	Australia	1	75	F (1)	Astrazeneca (1)	Acute Haemorrhagic Necrotizing Encephalopathy (1)	Died (1)
Sivji et al., [214]	2022	India	1	49	F (1)	Astrazeneca (1)	Acute Disseminated Encephalomyelitis (1)	Discharged (1)
Sluyts et al., [215]	2022	Belgium	1	48	M (1)	Moderna (1)	Vaccine-induced Encephalitis (1)	Discharged (1)
Sobh et al., [216]	2022	Saudi Arabia	1	23	F (1)	Astrazeneca (1)	Cerebral Vein Thrombosis (1)	Recovering (1)
Souza et al., [217]	2022	Australia	4	51-72	M (4)	Astrazeneca (4)	Inflammatory Demyelinating Polyneuropathy (4)	Discharged (3); Transferred to Rehabilitation Centre (1)
Sriwastava et al., [218]	2021	USA	3	41-67	M (2); F (1)	Pfizer (1); Moderna (1); Janssen (1)	Demyelinating Polyneuropathy (1); Not described (2)	Discharged (3)
Stefanou et al., [219]	2022	Greece	1	47	M (1)	Janssen (1)	Guillain Barre Syndrome and Fulminant encephalomyelitis (1)	Discharged (1)
Su et al., [220]	2022	Taiwan	1	39	F (1)	Astrazeneca (1)	Guillain Barre Syndrome (1)	Waiting to be transferred to Rehabilitation Centre (1)
Suri et al., [221]	2021	India	1	47	M (1)	Astrazeneca (1)	Acute Onset Inflammatory Demyelinating Polyneuropathy (1)	Discharged (1)
Syed et al., [222]	2021	USA	1	45	M (1)	Moderna (1)	Cerebral Venous Sinus Thrombosis (1)	Discharged (1)
Tabatabaee et al., [223]	2022	Iran	3	32-46	M (3)	Astrazeneca (1); Sinopharm (2)	Acute Motor Axonal Neuropathy subtype of Guillain Barre Syndrome (3)	Not described (3)
Tagliaferri et al., [224]	2021	USA	1	32	F (1)	Pfizer (1)	Multiple Sclerosis (1)	Discharged (1)
Takahashi et al., [225]	2022	Japan	1	65	F (1)	Pfizer (1)	Acute Inflammatory Demyelinating Polyneuropathy (1)	Transferred to Rehabilitation Centre (1)
Tan et al., [226]	2021	Malaysia	1	25	F (1)	Astrazeneca (1)	Longitudinal Extensive Transverse Myelitis (1)	Discharged (1)
Tapdia et al., [227]	2022	India	1	45	M (1)	Astrazeneca (1)	Frontal Lobe Syndrome (1)	Discharged (1)
Terreros-Caro et al., [228]	2021	Spain	1	50	M (1)	Pfizer (1)	Unilateral Bells Palsy (1)	Recovering (1)
Thant et al., [229]	2022	USA	1	66	M (1)	Janssen (1)	Guillain Barre Syndrome (1)	Recovering (1)
Tiede et al., [230]	2021	Germany	4	41-67	F (4)	Astrazeneca (4)	Cerebral Thromboembolic Events (4)	Recovering (4)
Toljan et al., [231]	2022	USA	5	29-46	M (2); F (3)	Pfizer (3); Moderna (2)	Multiple Sclerosis (5)	Transferred to Rehabilitation Centre (1); Not described (4)
Trimboli et al.,	2021	Italy	1	25	F (1)	Pfizer (1)	Guillain Barre Syndrome (1)	Transferred to Rehabilitation

[232]								Centre (1)
Vegezzi et al., [233]	2021	Italy	1	44	F (1)	Astrazeneca (1)	Myelitis (1)	Discharged (1)
Vitturi et al., [234]	2021	Italy	1	51	M (1)	Astrazeneca (1)	Parsonage Turner Syndrome (1)	Discharged (1)
Vogrig et al., [235]	2021	Italy	1	56	F (1)	Pfizer (1)	Acute Disseminated Encephalomyelitis (1)	Discharged (1)
Waheed et al., [236]	2021	USA	1	82	F (1)	Pfizer (1)	Guillain Barre Syndrome (1)	Transferred to Rehabilitation Centre (1)
Walter et al., [237]	2021	Germany	1	31	M (1)	Astrazeneca (1)	Thrombosis of Carotid Artery (1)	Recovering (1)
Yamakawa et al., [238]	2022	Japan	1	30	M (1)	Pfizer (1)	Miller Fisher Syndrome (1)	Discharged (1)
Yazdanpanah et al., [239]	2022	Iran	1	37	M (1)	Sinopharm (1)	Acute Disseminated Encephalomyelitis (1)	Discharged (1)
Yu et al., [240]	2021	China	1	36	F (1)	Sinovac (1)	Bells Palsy and Keratoconjunctivitis (1)	Discharged (1)
Zavala-Jonguitud et al., [241]	2021	Mexico	1	89	M (1)	Pfizer (1)	Delirium (1)	Discharged (1)
Zavari et al., [242]	2021	Iran	1	26	F (1)	Astrazeneca (1)	Aseptic Meningitis (1)	Discharged (1)
Zlotnik et al., [243]	2022	Israel	1	48	M (1)	Pfizer (1)	Anti-LGI1 Encephalitis (1)	Discharged (1)
Zubair et al., [244]	2022	USA	1	30	F (1)	Janssen (1)	Facial Diplegia Subtype of Guillain Barre Syndrome (1)	Discharged (1)
Zuhorn et al., [245]	2021	Germany	3	21-63	M (1); F (2)	Astrazeneca (3)	Encephalitis (3)	Discharged (3)
